# Comparative genomics reveals insight into the phylogeny and habitat adaptation of novel *Amycolatopsis* species, an endophytic actinomycete associated with scab lesions on potato tubers

**DOI:** 10.3389/fpls.2024.1346574

**Published:** 2024-03-27

**Authors:** Thippawan Wannawong, Wuttichai Mhuantong, Pipat Macharoen, Nantawan Niemhom, Jaruwan Sitdhipol, Neungnut Chaiyawan, Sarinna Umrung, Somboon Tanasupawat, Nakarin Suwannarach, Yukihiro Asami, Nattakorn Kuncharoen

**Affiliations:** ^1^ Department of Plant Pathology, Faculty of Agriculture, Kasetsart University, Bangkok, Thailand; ^2^ Food Biotechnology Research Team, Functional Ingredients and Food Innovation Research Group, National Center for Genetic Engineering and Biotechnology, National Science and Technology Development Agency, Pathum Thani, Thailand; ^3^ Enzyme Technology Research Team, Biorefinery and Bioproducts Technology Research Group, National Center for Genetic Engineering and Biotechnology, National Science and Technology Development Agency, Pathum Thani, Thailand; ^4^ Microbiological and Molecular Biological Laboratory, Scientific Instruments Center, School of Science, King Mongkut’s Institute of Technology Ladkrabang, Bangkok, Thailand; ^5^ Biodiversity Research Centre, Research and Development Group for Bio-Industries, Thailand Institute of Scientific and Technological Research, Pathum Thani, Thailand; ^6^ Department of Biochemistry and Microbiology, Faculty of Pharmaceutical Sciences, Chulalongkorn University, Bangkok, Thailand; ^7^ Center of Excellence in Microbial Diversity and Sustainable Utilization, Chiang Mai University, Chiang Mai, Thailand; ^8^ Department of Biology, Faculty of Science, Chiang Mai University, Chiang Mai, Thailand; ^9^ Graduate School of Infection Control Sciences, Kitasato University, Tokyo, Japan; ^10^ Ōmura Satoshi Memorial Institute, Kitasato University, Tokyo, Japan

**Keywords:** *Amycolatopsis*, biosynthetic gene cluster, comparative genomics, endophytic actinomycetes, potato scabby tuber, pathogenicity

## Abstract

A novel endophytic actinomycete, strain MEP2-6^T^, was isolated from scab tissues of potato tubers collected from Mae Fag Mai Sub-district, San Sai District, Chiang Mai Province, Thailand. Strain MEP2-6^T^ is a gram-positive filamentous bacteria characterized by *meso*-diaminopimelic acid in cell wall peptidoglycan and arabinose, galactose, glucose, and ribose in whole-cell hydrolysates. Diphosphatidylglycerol, phosphatidylglycerol, phosphatidylethanolamine, and hydroxy-phosphatidylethanolamine were the major phospholipids, of which MK-9(H_6_) was the predominant menaquinone, whereas iso-C_16:0_ and iso-C_15:0_ were the major cellular fatty acids. The genome of the strain was 10,277,369 bp in size with a G + C content of 71.7%. The 16S rRNA gene phylogenetic and core phylogenomic analyses revealed that strain MEP2-6^T^ was closely related to *Amycolatopsis lexingtonensis* NRRL B-24131^T^ (99.4%), *A. pretoriensis* DSM 44654^T^ (99.3%), and *A. eburnea* GLM-1^T^ (98.9%). Notably, strain MEP2-6^T^ displayed 91.7%, 91.8%, and 87% ANIb and 49%, 48.8%, and 35.4% dDDH to *A. lexingtonensis* DSM 44653^T^ (=NRRL B-24131^T^), *A. eburnea* GLM-1^T^, and *A. pretoriensis* DSM 44654^T^, respectively. Based on phenotypic, chemotaxonomic, and genomic data, strain MEP2-6^T^ could be officially assigned to a novel species within the genus *Amycolatopsis*, for which the name *Amycolatopsis solani* sp. nov. has been proposed. The type of strain is MEP2-6^T^ (=JCM 36309^T^ = TBRC 17632^T^ = NBRC 116395^T^). *Amycolatopsis solani* MEP2-6^T^ was strongly proven to be a non-phytopathogen of potato scab disease because stunting of seedlings and necrotic lesions on potato tuber slices were not observed, and there were no core biosynthetic genes associated with the BGCs of phytotoxin-inducing scab lesions. Furthermore, comparative genomics can provide a better understanding of the genetic mechanisms that enable *A. solani* MEP2-6^T^ to adapt to the plant endosphere. Importantly, the strain smBGCs accommodated 33 smBGCs encoded for several bioactive compounds, which could be beneficially applied in the fields of agriculture and medicine. Consequently, strain MEP2-6^T^ is a promising candidate as a novel biocontrol agent and antibiotic producer.

## Introduction

The genus *Amycolatopsis* belongs to the phylum *Actinomycetota*, class *Actinomycetia* (Oren and Garrity, 2021), order *Pseudonocardiales*, and the family *Pseudonocardiaceae*. This species was first proposed by Lechevalier et al. (1986) as *Amycolatopsis orientalis*. The taxonomic description of this genus was amended based on the 16S rDNA, chemotaxonomic characteristics, and genome sequences by Lee (2009); Tang et al. (2010), and Nouioui et al. (2018). Most species in the genus *Amycolatopsis* form long chains of substrate and aerial hyphae, which may differentiate into chains of squarish to oval fragments as spore-like structures. These characteristics constitute key morphological characteristics (Lechevalier et al., 1986). This genus is mycolate-less and contains *meso*-diaminopimelic acid in the peptidoglycan wall (Lechevalier and Lechevalier, 1970; Embley et al., 1988). Arabinose and galactose are diagnostic sugars found in whole-cell hydrolysates. The predominant isoprenoid quinone observed in *Amycolatopsis* is MK-9(H_4_) with phosphatidylethanolamine as a diagnostic phospholipid (Lechevalier et al., 1977). The G + C content in the genome of *Amycolatopsis* species is generally within the range of 66 to 75 mol% (Li et al., 2021). At the time of writing, the genus *Amycolatopsis* included 87 species with validly published names (Parte et al., 2020, https://lpsn.dsmz.de/genus/amycolatopsis).

Numerous species of the genus *Amycolatopsis* have adapted to occupy many diverse biological niches, such as soil (Camas et al., 2013), plants (Mingma et al., 2020), salt mines (Tatar et al., 2013), lakes (Li et al., 2021), marine environments (Bian et al., 2009), insects (Beemelmanns et al., 2017), animals (Labeda et al., 2003), and humans (Huang et al., 2004). The *planta Amycolatopsis* species have been discovered inside the tissues of different plant species: *A. samaneae* recovered from the roots of *Samanea saman* (Duangmal et al., 2011), and *A. jiangsuensis* was isolated from the stems of *Dendranthema indicum* (Xing et al., 2013), *A. stemonae* isolated from the stems of *Stemona* sp. (Klykleung et al., 2015), *A. anabasis* isolated from the roots of *Anabasis elatior* (Wang et al., 2020), and *A. dendrobii* isolated from the roots of *Dendrobium heterocarpum* Lindl (Tedsree et al., 2021). However, their ability to adapt to plants is not fully understood.

Members of the genus *Amycolatopsis* are closely connected with a history of antibiotic drug discovery as invaluable commercial producers of secondary metabolites with antibacterial, antifungal, or antiviral activities. They have continued to gain considerable attention in the search for new drugs (Kisil et al., 2021). Metabolites are synthesized by several diverse gene clusters in the genome, called biosynthetic gene clusters (BGCs). Fortunately, the reduced costs of whole-genome sequencing and public access to genome databases are now allowing the scientific community to sequence and share thousands of prokaryotic genomes worldwide. This can help to comprehensively detect and compare BGCs between organisms, as well as to identify their chemical structures using robust bioinformatics tools (Alam et al., 2022), such as antiSMASH 7.0 (Blin et al., 2023), BAGEL4 (van Heel et al., 2018), and PRISM 4 (Skinnider et al., 2020). This knowledge and these tools can guide us in predicting where to find promising new compounds and help us determine if the search for new producers should be based on phylogeny, geography, or specific ecological niches (Adamek et al., 2018).

In the present study, the novel endophytic *Amycolatopsis* strain MEP2-6^T^ was isolated from scab lesions on potato tuber surfaces; however, there have been no reports of its association with potato scab disease. Thus, we aimed to comprehensively characterize *Amycolatopsis* strain MEP2-6^T^ based on polyphasic and genome-based taxonomy and evaluate the pathogenicity of strain MEP2-6^T^ in plants. In addition, we determine how this strain can adapt to reside in plants, study the diversity of the secondary metabolite biosynthetic gene clusters (smBGCs) of the strain MEP2-6^T^, and compare the distribution pattern of smBGCs with its closest type strains. This work represents the first of its kind to report on the *Amycolatopsis* species inhabiting potato scab lesions and can provide a better understanding of the genetic mechanisms of this species that occupy plants. We also reported the genetic potential of this species to produce various types of secondary metabolites. These outcomes can contribute to the identification of specialized biosynthetic pathways that are of particular interest and serve as a guide for antibiotic drug discovery.

## Materials and methods

### Bacterial isolation and preservation

Scabby tubers of potato (*Solanum tuberosum* L.) (**Supplementary Figure 1**) collected from Mae Fag Mai Sub-district, San Sai District, Chiang Mai Province, northern Thailand (18○58’56.3’’N, 98○58’45.8’’E), were washed with running tap water and then surface sterilized according to the method described by Kuncharoen et al. (2018) with slight modifications. Briefly, the scab-infected potato tubers were washed with running tap water to remove soil particles, soaked in 75 % (*v/v*) ethanol for 1 min, drenched in 1 % (*v/v*) NaOCl for 3 min, rinsed three times in sterile distilled water, and air-dried in a laminar flow. Single lesions appearing at the border between healthy and scab tissues of the surface-sterilized tubers were aseptically cut into small pieces, homogenized using a sterile mortar with 1 ml of 0.85% (*w/v*) NaCl, and incubated in a water bath at 60°C for 10 min to eliminate any competing rhizobacteria (Fyans et al., 2016). The homogenate was diluted 100-fold. Subsequently, 0.1 ml was spread onto 2.5% (*w/v*) water agar (Arai, 1975) supplemented with 25 µg nalidixic acid ml^−1^ and 50 µg cycloheximide ml^−1^ and then incubated at 30°C in the dark for 14 days. An interesting colony of strain MEP2-6^T^ was isolated and purified on International *Streptomyces* Project medium 2 (ISP 2) agar (Shirling and Gottlieb, 1966), maintained on ISP 2 agar slant, stored at −20°C and −80°C in the ISP 2 broth supplemented with 20 % (*v/v*) glycerol, and lyophilized for long-term preservation.

### Genomic DNA extraction

Genomic DNA of strain MEP2-6^T^ was extracted using the FavorPrepTM Tissue Genomic DNA Extraction Mini Kit (Favorgen, Taiwan), according to the manufacturer’s instructions. DNA quality and quantity were determined using 1% (*w/v*) agarose gel electrophoresis and NanoDrop spectrophotometer (Thermo Fisher Scientific, Waltham, MA, USA). The purified genomic DNA was stored at −20°C until use.

### Genome sequencing, assembly, and annotation

The genome sequence of strain MEP2-6^T^ was successfully constructed by combining Illumina HiSeq 2,500 paired-end sequencing (Illumina Inc., San Diego, CA, USA) at Novogene (Biopolis Way, Singapore) and the GridION sequencer (Oxford Nanopore Technologies—ONT, UK) at Siriraj Long-read Lab (Siriraj Medical Research Center, Thailand). The genome assembly consists of four steps: first, raw Illumina reads were performed the quality control by removing low-quality sequences and trimming the adapter and primer sequences using fastp v0.20.0 (Chen et al., 2018), and then evaluated their sequence quality with FastQC v0.12.0 (https://github.com/s-andrews/FastQC) and MultiQC v1.17 (Ewels et al., 2016); second, ONT raw signals were base called and demultiplexed using Guppy v3.4.5 (ONT) with the use of a specific high-accuracy model (-c dna_r9.4.1_450bps_hac.cfg) to obtain raw ONT reads; third, the raw ONT long reads were filtered by their quality and sequence length using the Nanofilt program (De Coster et al., 2018), and then adapters were trimmed using Porechop v0.2.4 (https://github.com/rrwick/Porechop). Finally (step 4), the cleaned short and long sequences were assembled *de novo* using SPAdes v3.15.4 (Antipov et al., 2016; Prjibelski et al., 2020) with a minimum contig size of 500 bps.

The assembled genome of strain MEP2-6^T^ and the publicly available genome assemblies of the closely related *Amycolatopsis* species, as well as an outgroup with validly published names downloaded from the NCBI database (3 June 2022) using the E-utilities Command (Kans, 2022), were estimated for genome completeness and contamination using CheckM v1.1.6 (Parks et al., 2015). Contiguity and the completeness of universal single-copy orthologs were assessed using QUAST v5.2.0 (Gurevich et al., 2013) and BUSCO v5.4.7 (Simão et al., 2015; Manni et al., 2021).

The genome of strain MEP2-6^T^ was annotated using Rapid Annotation with the Subsystem Technology (RAST) server (Aziz et al., 2008) using the RASTtk algorithm (Brettin et al., 2015) and re-annotated according to the NCBI Prokaryotic Genome Annotation Pipeline (PGAP) (Tatusova et al., 2016). The genomes of the closest *Amycolatopsis* species were obtained from NCBI GenBank and annotated using the RAST server. The accession no. of the genomes are shown in **Table 1**.

**Table 1:** Genome features of strain MEP2-6^T^ and its closest type strains.

Feature	1	2	3	4
**Accession no.**	JAWQJT000000000	JADBEG000000000	RSEC00000000	FNUJ00000000
**Genome size (bp)**	10,277,369	10,737,921	10,230,128	10,299,026
**GC content (mol%)**	71.7	71.5	71.8	71.2
**Assembly level**	Scaffold	Scaffold	Scaffold	Scaffold
**Number of contigs**	4	1	74	31
**Total genes**	9,374	10,241	9,495	9,894
**Protein coding genes**	9,261	9,827	9,211	9,732
**5S/16S/23S rRNA**	3/2/2	2/2/5	5/5/16	5/5/1
**tRNAs**	50	51	53	52
**Total pseudogenes**	51	414	202	96

Genomes: 1, Strain MEP2-6^T^; 2, *A. lexingtonensis* DSM 44653^T^; 3, *A. eburnea* GLM-1^T^; 4, *A. pretoriensis* DSM 44654^T^.

### Analysis of 16S rRNA gene sequence

PCR amplification of the 16S rRNA gene was performed as described by Suriyachadkun et al. (2009). The purified PCR product of the 16S rRNA gene was sequenced by Macrogen (Seoul, Republic of Korea) using universal primers, as previously described by Lane (1991). The sequence was trimmed manually using the BioEdit software (Hall, 1999) to obtain an almost complete 16S rRNA gene sequence (1,407 bp). The sequence was aligned with the sequences of available valid type strains in the genus *Amycolatopsis*, and any sequence similarities on the EzBioCloud server (https://www.ezbiocloud.net/) were determined (Yoon et al., 2017).

### 16S rRNA gene and genome phylogenies

Phylogenetic trees based on the 16S rRNA gene sequence were generated using the neighbor-joining (Saitou and Nei, 1987), maximum-likelihood (Felsenstein, 1981), and maximum-parsimony (Fitch, 1972) tree-making methods using MEGA X (Kumar et al., 2018). Evolutionary distance matrices were computed based on Kimura’s two-parameter model (Kimura, 1980). The confidence of the tree topologies was statistically evaluated using 1,000 bootstrap resampling replicates (Felsenstein, 1985).

OrthoFinder v2.5.4 (Emms and Kelly, 2017, 2018, 2019) was used to construct species phylogeny based on 1,104 orthologs found in genomes of strain MEP2-6^T^ and its 19 closely related *Amycolatopsis* species within an outgroup, *Streptomyces scabiei* 87.22^T^. The genome phylogeny was then visualized using the interactive Tree of Life (iTOL) (Letunic and Bork, 2019) and further modified using Inkscape (https://inkscape.org/).

### Phenotypic characterization

Cell morphology of strain MEP2-6^T^ was observed using scanning electron microscopy (JEOL, JSM-IT500HR, Tokyo, Japan) after being grown on yeast malt extract agar (ISP 2) at 30°C for 21 days. The cultural characteristics of strain MEP2-6^T^ and its closest type strains were determined after 14 days of incubation at 30°C on various agar media: ISP 2 agar, oatmeal agar (ISP 3), inorganic salt-starch agar (ISP 4), glycerol-asparagine agar (ISP 5), peptone-yeast extract iron agar (ISP 6), tyrosine agar (ISP 7), and nutrient agar (NA) (Shirling and Gottlieb, 1966). The color of the colonies and diffusible pigments were assigned based on the ISCC-NBS color system (Kelly, 1964). Growth at different temperatures (15°C, 25°C, 30°C, 37°C, 45°C, and 50°C) and tolerance levels to NaCl (1%–10%, *w/v*) were assessed using ISP 2 agar as the basal medium, and the effect of pH on growth ranging from 4 to 10 (at intervals of 1 pH unit) was examined in ISP 2 broth at 30°C for 14 days using the following buffer system: acetate buffer (pH 4–5), phosphate buffer (pH 6–8), and glycine–sodium hydroxide buffer (pH 9–10). Utilization of carbohydrates as the sole carbon source was observed using ISP 9 as the basal medium supplemented with a final concentration of 1% (*w/v*) of the carbon sources (Shirling and Gottlieb, 1966). Acid production from carbohydrates was determined using a basal inorganic nitrogen medium, according to the method described by Gordon et al. (1974). Starch hydrolysis, nitrate reduction, milk peptonization, milk coagulation, gelatin liquefaction, and H_2_S production were assessed on ISP 4 agar, ISP 8 broth (0.5% peptone, 0.3% beef extract, and 0.1% KNO3, pH 7.0), 10% (*w/v*) skimmed milk agar, 10% (*w/v*) skimmed milk broth, glucose-peptone-gelatin medium (2.0% glucose, 0.5% peptone, and 20% gelatin, pH 7.0), and ISP 6 agar, respectively. Enzymatic activity was assayed using the API ZYM (bioMérieux) commercial kit, according to the manufacturer’s instructions.

### Chemotaxonomic characterization

Freeze-dried whole cells of strain MEP2-6^T^ and its closest type strains for chemotaxonomic analyses were obtained after growth in yeast extract dextrose broth (1% yeast extract, 1% dextrose, pH 7.0) at 30°C (200 rpm) for 7 days. Isomers of cell wall diaminopimelic acid (A_2_pm) and reducing sugars of strain MEP2-6^T^ whole-cell hydrolysates were determined using thin-layer chromatography (TLC) (Staneck and Roberts, 1974). The *N*-acyl group of muramic acid in the cell wall peptidoglycan of strain MEP2-6^T^ was examined according to the method described by Uchida and Aida (1984). The cellular phospholipids of strain MEP2-6^T^ were extracted and identified using 2-dimensional TLC, as previously described by Minnikin et al. (1984). Mycolic acid was extracted and monitored by TLC following the method described by Tomiyasu (1982). Cellular fatty acid methyl esters of all strains were prepared according to the method described by Sasser (1990) and analyzed using gas chromatography (MIDI, Sherlock Microbial Identification System, TSBA6 Sherlock Version 6.2B, USA). Isoprenoid quinones were extracted using the method previously employed by Collins et al. (1977) and were detected by LC-DAD-ESI-MS (AB Sciex, Framingham, MA, USA) equipped with a CAPCELL CORE C18 column (3.0 mm i.d. × 100 mm), OSAKA SODA Co., Ltd., using methanol–isopropanol (7:3, *v/v*). Finally, UV detection was performed at 270 nm wavelength.

### Comparative genomic analyses

The genome of strain MEP2-6^T^ was used to determine the taxonomic parameters between its closely related strains. This process involved average nucleotide identity based on BLAST (ANIb) and MUMmer (ANIm), and digital DNA–DNA hybridization (dDDH) values using JSpeciesWS (Richter et al., 2016) and the Genome-to-Genome Distance Calculator (GGDC) 3.0, with the recommended formula 2 (Meier-Kolthoff et al., 2013, 2022), respectively, to verify its taxonomic status. OrthoVenn3 (Sun et al., 2023, https://orthovenn3.bioinfotoolkits.net) was used to analyze shared and strain-specific orthologous clusters. A cluster of orthologous genes (COGs) in the unique orthologous cluster was functionally annotated and identified using eggNOG v5.0 (Huerta-Cepas et al., 2019), a database of orthology relationships, functional annotations, and gene evolutionary histories, as well as the Kyoto Encyclopedia of Genes and Genomes (KEGG) database (Kanehisa et al., 2016), a database resource used to gain an in-depth understanding of the high-level functions and utilities of the biological system. AntiSMASH 7.0 (Blin et al., 2023) was used to identify secondary metabolite biosynthetic gene clusters (smBGCs) with default settings. Gene clusters with a BLAST identity of >80% were determined to belong to the same smBGCs. The results were collected in a presence/absence matrix to establish the number of genes designed for individual smBGCs in each bacterial strain. Hierarchical cluster analysis using the DICE coefficient with Unweighted Pair Group Method with Arithmetic (UPGMA) mean value was implemented with PAST (Hammer et al., 2001).

### Plant-pathogenicity test

Strain MEP2-6^T^ was examined for pathogenicity using the plant seedling method previously described by Flores-Gonzalez et al. (2008) and Dees et al. (2013), with slight modifications. Cherry tomato seeds (*Solanum lycopersicum* var. *cerasiforme*) were surface-disinfected with 6% (*v/v*) NaOCl for 10 min, rinsed three times with sterile distilled water, placed on sterile tissue paper, and air-dried under laminar flow. Subsequently, sterilized seeds were aseptically placed on an 8-day-old culture of strain MEP2-6^T^ grown on ISP 2 agar medium and incubated at room temperature for 8 days. *Streptomyces scabiei* WSLK1-9 and ISP 2 agar plates without bacteria were used as controls. The appearance of seedlings after bacterial growth was recorded. The strain was considered phytopathogenic if the seeds displayed stunting or did not germinate. This experiment was performed in triplicate. A potato tuber slice technique was also used to determine the pathogenicity of strain MEP2-6^T^ following the procedure previously described by Loria et al. (1995) and Henao et al. (2021), with slight modifications. The potato tubers cv. Spunta were surface-sterilized in 3% (*v/v*) NaOCl for 5 min, rinsed three times with sterile distilled water to eliminate the disinfectant, and air-dried under laminar flow. The surface-sterilized tubers were cut into slices (0.5 cm thick) and placed on moist sterile membrane filter paper in sterile Petri dishes. Strain MEP2-6^T^ and *S. scabiei* WSLK1-9 were grown on ISP2 agar medium for 8 days at 30°C to produce high mycelia and spore masses. Agar plugs of the sporulated strain MEP2-6^T^ were flipped and placed at the center (pith tissue) of potato slices. The agar plugs of *Streptomyces scabiei* WSLK1-9 and ISP 2 without bacteria were used as controls. The moist Petri dishes were incubated in the dark at 30°C for six days. Each isolate was tested in triplicate.

### *In planta* colonization of *Solanum tuberosum* L. cv. Spunta and microscopic observation

The axillary buds of sprouted potato (*S. tuberosum* L. cv. Spunta) were excised and surface sterilized by soaking in 10% and 5% (*v/v*) NaClO solution for 15 min and 10 min, respectively, and rinsed twice thoroughly with sterile distilled water to eliminate the sterilizing agent (Barker, 1953). The sterilized buds were dried in a flow hood, aseptically cut into small pieces, placed on Murashige–Skoog agar media supplemented with 0.1% (*w/v*) activated charcoal (Murashige and Skoog, 1962; Feyissa et al., 2005), and incubated at 25°C under 1,000 lx illumination using 40-watt TL 33 Philips fluorescent lamps for 14 days (Roca et al., 1978). The prepared 8-day-old strain MEP2-6^T^ agar plug was directly applied at the wounding (pin-prick) stem node site of 14-day-old *S. tuberosum* L. cv. Spunta cultures and incubated at 25°C under 1,000 lx illumination for five days. Colonization was monitored five days post-inoculation using a bright-field microscopic technique (Thomas and Reddy, 2013). A ZEISS Axiolab 5 optical microscope, together with the microscope camera Axiocam 208 color and ZEISS Labscope Imaging App v4.2.1 (Carl Zeiss Microscopy Deutschland GmbH, Oberkochen, Germany) was used for bright-field microscopy. Thin stem tissues were prepared using a free-hand-cut sectioning technique with a fine razor blade. The tissues were examined after mounting with Shear’s mounting medium (6.0 g potassium acetate, 120 ml glycerol, 180 ml 95% (*v/v*) ethanol, and 300 ml distilled water) on acetone-washed and autoclaved microscope slides under oil immersion (×100). Images were captured with the ZEISS Labscope Imaging App and processed using Adobe Photoshop 2021 v22.5.9.1101 (Adobe Systems Inc., San Jose, CA, USA) software.

### Determination of extracellular carbohydrate-degrading enzyme production

Strain MEP2-6^T^ and its closest strains were investigated for their production of extracellular carbohydrate-degrading enzymes, including endoglucanase, chitinase, and pectinase, according to the method described by Kumla et al. (2020). An agar mycelial plug of each strain was inoculated into the tested agar media. Endoglucanase, chitinase, and pectinase production were investigated on carboxymethyl cellulose (CMC) agar, colloidal chitin agar, and pectin agar, respectively. The plates were incubated at 25°C in the dark for 5 days. Colonies of all strains were immersed in 1% (*w/v*) Congo red for 30 min and then rinsed with 1 M NaCl for 15 min for chitinase and endoglucanase tests. For the pectinase test, colonies of all strains were immersed in 1% (*w/v*) hexadecyltrimethylammonium bromide for 15 min and rinsed with sterile deionized (DI) water. A positive result for the production of each enzyme was indicated by the presence of a clear transparent zone around the colony. Four replicates were performed for each enzyme.

## Results

### Sequence analysis and 16S rRNA gene and genome phylogenies

**Figure 1:**
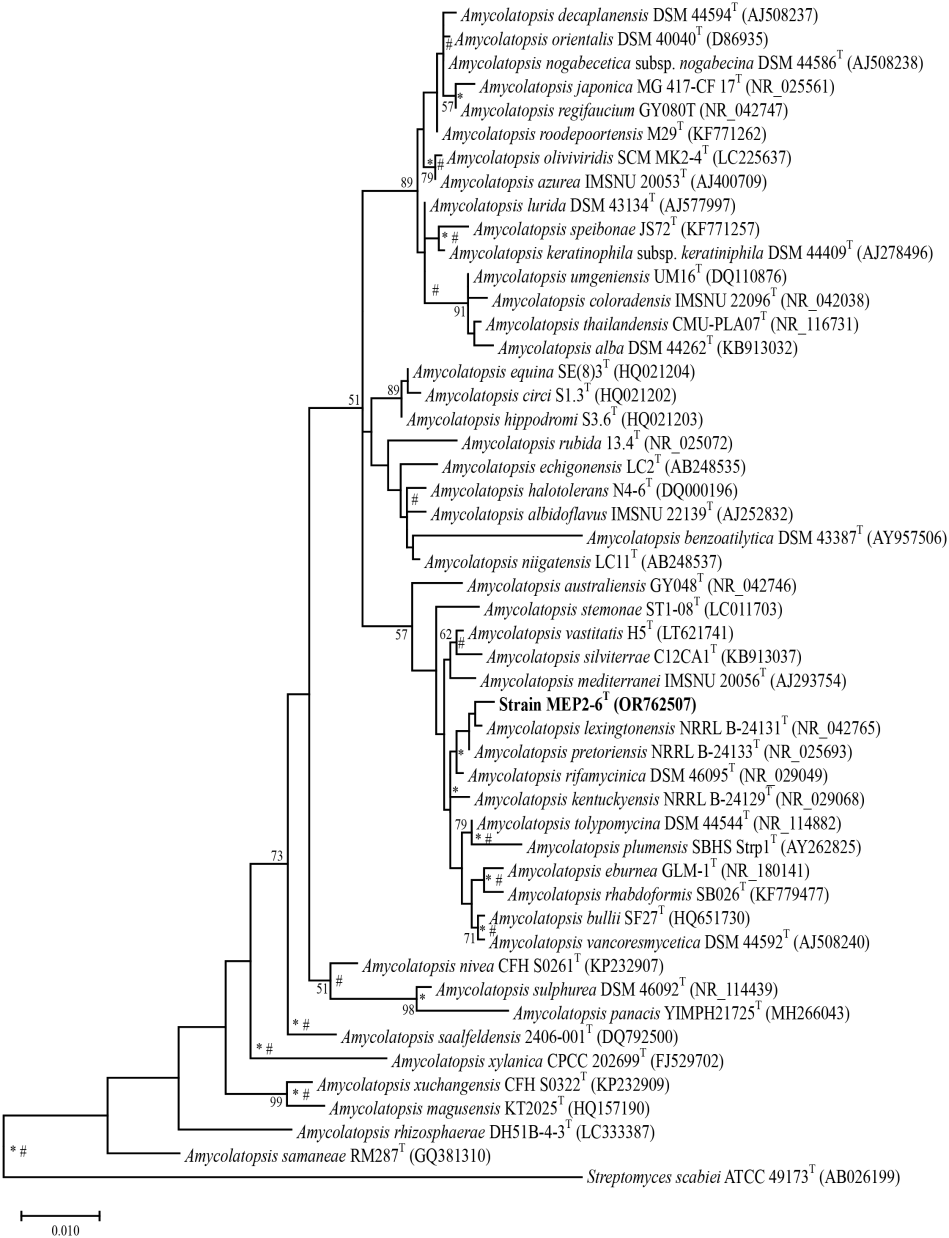
Maximum-likelihood phylogenetic tree based on the 16S rRNA gene sequences of strain MEP2-6^T^ and its closely related type strains with validly published names. *Streptomyces scabiei* 87.22^T^ was used as the outgroup. Asterisks and sharps (*, #) indicate that the corresponding nodes were also recovered in the neighbor-joining and maximum-parsimony trees, respectively. Bootstrap values of ≥50% (percentages of 1,000 replications) are shown at branch nodes Bar, 0.01 substitutions per nucleotide position.

**Figure 2:**
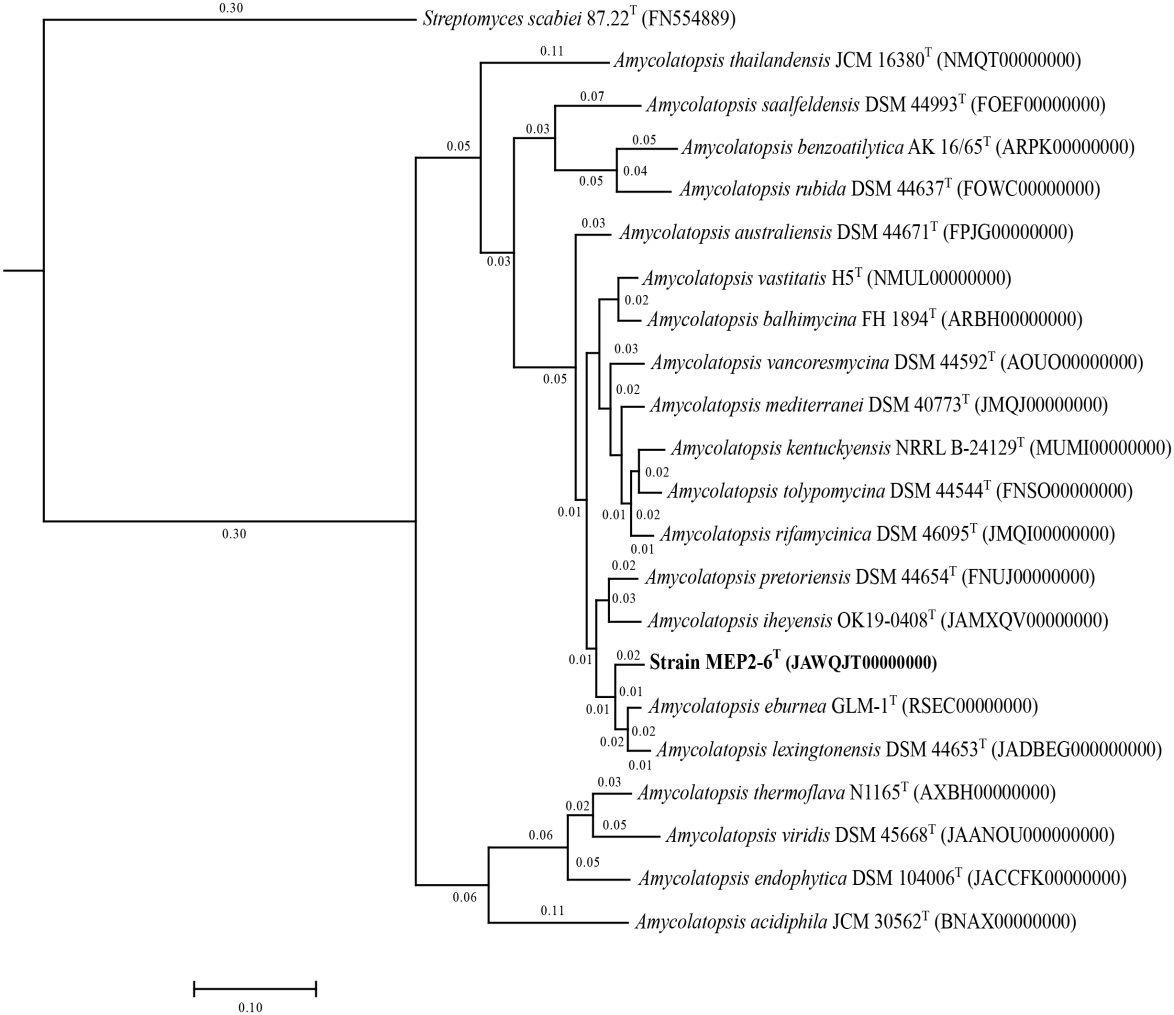
A core phylogenomic tree based on 1,104 orthologous proteins illustrates the evolutionary relationship between strain MEP2-6^T^ and its closely related *Amycolatopsis* species. *Streptomyces scabiei* 87.22^T^ was used as the outgroup. The distance matrices between species are shown at the node.

16S rRNA gene sequence analysis revealed that strain MEP2-6^T^ (1,407 bp, accession no. OR762507) is a member of the genus *Amycolatopsis*, the order *Pseudonocardiales*, and the family *Pseudonocardiaceae*. Sequence analysis revealed that strain MEP2-6^T^ shared a close relationship with *Amycolatopsis lexingtonensis* NRRL B-24131^T^, *A. pretoriensis* DSM 44654^T^, *A. kentuckyensis* NRRL B-24129^T^, *A. rifamycinica* DSM 46095^T^, *A. tolypomycina* DSM 44544^T^, and *A. eburnea* GLM-1^T^, with sequence similarities of 99.4% (9 nt difference at 1,400), 99.3% (10 nt difference at 1,401), 99.0% (14 nt difference at 1,400), 99.1% (12 nt difference at 1,400), 99.1% (12 nt difference at 1,400), and 98.9% (15 nt difference at 1,400), respectively. A maximum-likelihood phylogenetic tree based on the 16S rRNA gene sequence (**Figure 1**) indicated that strain MEP2-6^T^ formed a tightly independent cluster with the closest species, *A. lexingtonensis* NRRL B-24131^T^ and *A. pretoriensis* DSM 44654^T^. The clusters that could be recovered in neighbor-joining and maximum parsimony trees are presented in **Supplementary Figures 2**, **3**, respectively. Genome phylogeny (**Figure 2**) suggested that strain MEP2-6^T^ formed a robustly liberated clade with *A. lexingtonensis* DSM 44653^T^ (=NRRL B-24131^T^) and *A. eburnea* GLM-1^T^. Therefore, based on a combination of sequence analysis, 16S rRNA gene phylogenetic tree, and phylogenomic tree, *A. lexingtonensis* NRRL B-24131^T^ (=JCM 12672^T^ =DSM 44653^T^), *A. pretoriensis* DSM 44654^T^ (=JCM 12673^T^), and *A. eburnea* GLM-1^T^ (=TBRC 9315^T^) were used to further clarify the phenotypic characteristics, chemotaxonomic properties, and genome comparisons.

### Phenotypic characteristics

**Figure 3:**
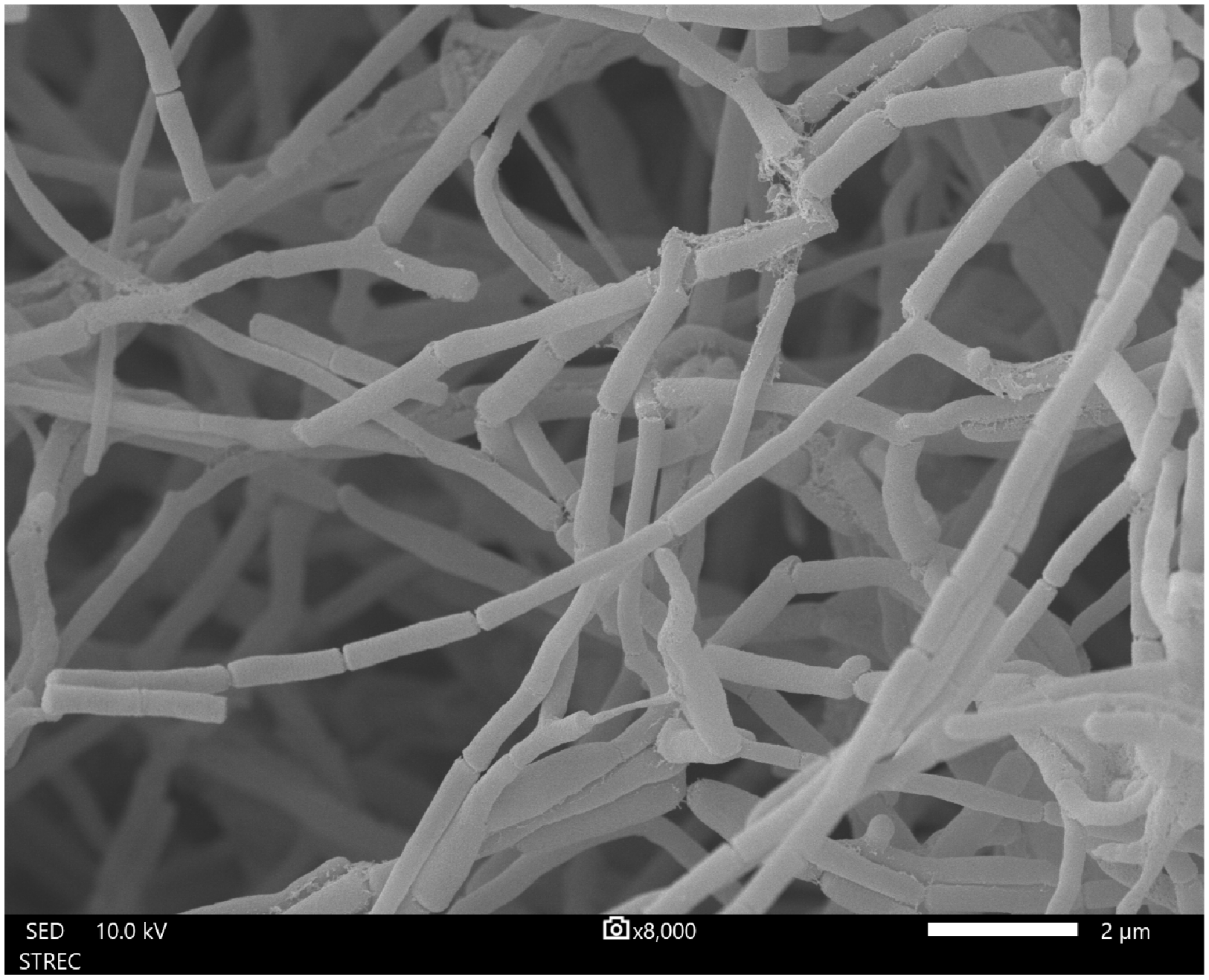
Scanning electron micrograph of strain MEP2-6^T^ grown on ISP 2 agar medium at 30°C for 14 days showing the morphology of the substrate and aerial mycelia. Bar, 2 µm.

**Figure 4:**
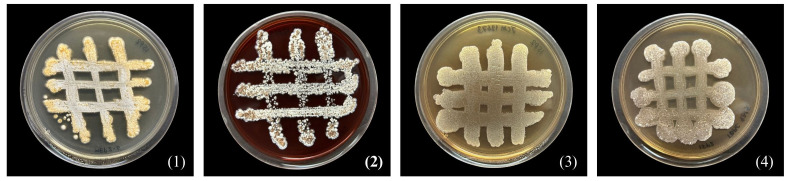
Differential colonial appearances of strain MEP2-6^T^ and its closest type strains grown on ISP 2 agar medium at 30°C for 14 days. Strain: 1, MEP2-6^T^; 2, *Amycolatopsis lexingtonensis* JCM 12672^T^; 3, *A. pretoriensis* JCM 12673^T^; 4, *A. eburnea* TBRC 9315^T^.

Strain MEP2-6^T^ produced septal substrate and aerial hyphae (0.4–0.5 µm × 1.1–1.4 µm in size) that fragmented into rod-like elements (**Figure 3**). The strain adequately developed moderate orange-colored substrate mycelia and pale orange yellow aerial mycelia on ISP 2 agar medium but did not form aerial hyphae on ISP 5 and nutrient agar media. Soluble pigments were not observed in any of the media tested. Strain MEP2-6^T^ also grew well on ISP 2, ISP 3, ISP 4, and ISP 7, whereas it grew moderately and/or poorly grew on ISP 5, ISP 6, and nutrient agar media. The cultural characteristics of strain MEP2-6^T^ and the phylogenetically related type strains on ISP 2 agar medium are shown in **Figure 4**, and other media are presented in **Supplementary Table 1**, **Supplementary Figure 4**. Growth occurred at 15°C–37°C (optimum, 30°C), pH 5–9 (optimum, 7), and 1%–4% (*w/v*) NaCl. Strain MEP2-6^T^ utilized amygdalin, L-arabinose, D-fructose, D-galactose, D-glucose, D-melezitose, *myo*-inositol, L-rhamnose, D-sucrose, and D-xylose as the sole carbon sources. The strain had the ability to reduce nitrate, coagulate, and peptonize milk, and to liquefy gelatin, but did not hydrolyze starch and produce H_2_S. In the API ZYM test, it was positive for leucine arylamidase, valine arylamidase, cystine arylamidase, α-chymotrypsin, and Naphthol-AS-BI-phosphohydrolase, while acid phosphatase, *N*-acetyl-*β*-glucosaminidase, and α–fucosidase were weakly positive. The phenotypic properties that distinguished strain MEP2-6^T^ from other closely related strains are listed in **Table 2**.

**Table 2:** Differential phenotypic characteristics between strain MEP2-6^T^ and its closely related strains.

Characteristic	1	2	3	4
**Color of aerial mycelia on ISP 2**	Pale orange yellow	Greenish white	Yellowish white	Yellowish white
**Color of substrate mycelia on ISP 2**	Moderate orange	Dark yellowish brown	Moderate orange yellow	Light yellow
**Color of soluble pigment in ISP 2**	−	Very dark red	−	−
**Growth on ISP 5**	Medium	Good	Good	Good
**Temperature range (°C)**	15–37	15–45	15–37	15–45
**pH range**	5–9	6–9	6–9	4–11
**NaCl range % (*w/v*)**	1–4	1–5	1–5	1–5
Utilization of:				
**Amygdalin**	+	+	−	−
**L-Arabinose**	+	+	−	+
**D-Melezitose**	w	+	+	+
Acid production from:				
**D-Sucrose**	+	w	+	−
**D-Xylose**	−	−	+	+
**Coagulation of milk**	+	−	−	+
**Peptonization of milk**	+	+	+	+
API ZYM				
**Acid phosphatase**	w	+	+	−
**Alkaline phosphatase**	−	+	+	−
**α-Chymotrypsin**	+	+	+	−
**Cystine arylamidase**	+	w	+	−
**Esterase (C4)**	−	−	+	−
**Esterase Lipase (C8)**	−	−	+	−
**α-Fucosidase**	w	−	w	+
**β-Galactosidase**	−	+	+	−
**α-Glucosidase**	−	−	+	+
**β-Glucosidase**	−	−	+	+
***N*-Acetyl-*β*-Glucosaminidase**	w	+	+	+
**Lipase (C14)**	−	−	+	−
**Leucine arylamidase**	+	+	+	−
**α-Mannosidase**	−	+	+	+
**Naphthol-AS-BI-Phosphohydrolase**	+	−	+	−
**Valine arylamidase**	+	−	w	−

Strain: 1, MEP2-6^T^; 2, *A. lexingtonensis* JCM 12672^T^; 3, *A. pretoriensis* JCM 12673^T^; 4, *A. eburnea* TBRC 9315^T^. All data are obtained from this study. +, Positive; −, negative; w, weakly positive.

### Chemotaxonomy

Strain MEP2-6^T^ comprises *meso*-diaminopimelic acid as the diagnostic diamino acid in the cell wall peptidoglycan. Arabinose, galactose, glucose, and ribose were identified as the diagnostic sugars in the whole-cell hydrolysate. The *N*-acyl group of muramic acid in peptidoglycan is acetyl, whereas mycolic acids are absent. The polar lipid profile consisted of diphosphatidylglycerol (DPG), phosphatidylglycerol (PG), phosphatidylethanolamine (PE), hydroxy-phosphatidylethanolamine (OH−PE), an unidentified aminophospholipid (APL), six unidentified phospholipids (PL1−PL6), an unidentified glycolipid (GL), and five unidentified lipids (L1−L5) (**Supplementary Figure 5**). The major menaquinone in strain MEP2-6^T^ was MK-9(H_6_) (90.1%), while MK-9(H_4_) (9.9%) was a minor component. The iso-C_16:0_ (37.9%) and iso-C_15:0_ (13.7%), which accounted for >10% of the total fatty acids, were the predominant cellular fatty acids in the strain profile. Differences in the types and quantities of cellular fatty acids of strain MEP2-6^T^ and its closely related species are presented in **Table 3**.

**Table 3:** Different cellular fatty acid profiles (%) of strain MEP2-6^T^ and its closely related type strains.

Fatty acid	1	2	3	4
Saturated fatty acid				
**C_14:0_**	0.5	−	−	1.0
**C_16:0_**	9.3	11.5	7.2	18.0
**C_17:0_**	3.3	4.6	3.7	3.1
**C_18:0_**	3.4	5.9	1.7	2.4
Unsaturated fatty acid				
**C_17:1_ *ω*6c**	–	–	3.7	–
**C_17:1_ *ω*8c**	1.6	1.3	1.6	0.7
**C_18:1_ *ω*9c**	0.8	0.7	–	–
Saturated branched fatty acids				
**iso-C_14:0_**	2.0	1.5	2.5	3.2
**iso-C_15:0_**	13.7	15.5	16.4	13.3
**anteiso-C_15:0_**	2.5	1.6	3.0	2.5
**iso-C_16:0_**	37.9	26.7	32.4	37.3
**iso-C_16:0_H**	0.5	–	–	–
**anteiso-C_16:0_**	0.7	1.4	0.6	–
**iso-C_17:0_**	4.8	5.2	5.6	3.2
**iso-C_17:0_ 3-OH**	–	0.6	–	–
**anteiso-C_17:0_**	9.0	10.9	10.8	5.8
**iso-C_18:0_**	–	0.6	–	–
10-Methyl fatty acids				
**10-methyl C1_7:0_**	–	1.1	0.7	–
**Summed feature 3^a^**	5.2	5.8	4.7	4.1
**Summed feature 9^b^**	2.0	2.2	1.8	1.9

Strain: 1, MEP2-6^T^; 2, *A. lexingtonensis* JCM 12672^T^; 3, *A. pretoriensis* JCM 12673^T^; 4, *A. eburnea* TBRC 9315^T^. All data are obtained from this study. The amount of fatty acid less than 0.5% in all strains was omitted. ^a^C_17:0_ω6c or C_16:1_ω6c, ^b^10-methyl C_16:0_-, absence.

### Genomic feature and comparison

The genome sequence of strain MEP2-6^T^ was 10,277,369 bp in size with a GC content of 71.74 mol% (accession no. JAWQJT000000000). The genomic features of the genome and the closest species of *Amycolatopsis* are summarized in **Table 1**. Pairwise genome-level comparisons between strain MEP2-6^T^ and its phylogenetically closest relatives, including ANIb, ANIm, and dDDH values, were calculated to accurately delineate the species (**Table 4**). The strains exhibited 91.7%, 91.8%, and 87% ANIb and 93%, 92.9%, and 86% ANIm to *A. lexingtonensis* DSM 44653^T^ (=JCM 12672^T^), *A. eburnea* GLM-1^T^ (=TBRC 9315^T^), and *A. pretoriensis* DSM 44654^T^ (=JCM 12673^T^), respectively. The dDDH values for the comparison of strain MEP2-6T to *A. lexingtonensis* DSM 44653^T^, *A. eburnea* GLM-1^T^, and *A. pretoriensis* DSM 44654^T^ were 49%, 48.8%, and 35.4%, respectively. Both values were significantly lower than the threshold values of 95%–96% ANI (Richter and Rosselló-Móra, 2009) and 70% dDDH (Wayne et al., 1987; Goris et al., 2007), which is recommended for use in species discrimination. Consequently, strain MEP2-6^T^ can be officially recognized as a novel species within the genus *Amycolatopsis*.

**Table 4:** ANIb, ANIm, and dDDH values between strain MEP2-6^T^ and its closest *Amycolatopsis* species.

Query genome	Reference genome	%ANIb	%ANIm	%dDDH (formula 2)	Model C.I.
**1**	**2**	91.7	93.0	49.0	[46.4%–51.6%]
**1**	**3**	91.8	92.9	48.8	[46.2%–51.4%]
**1**	**4**	87.0	86.5	35.4	[33.0%–37.9%]

Genomes: 1, Strain MEP2-6^T^; 2, *A. lexingtonensis* DSM 44653^T^; 3, *A. eburnea* GLM-1^T^; 4, *A. pretoriensis* DSM 44654^T^.

**Figure 5:**
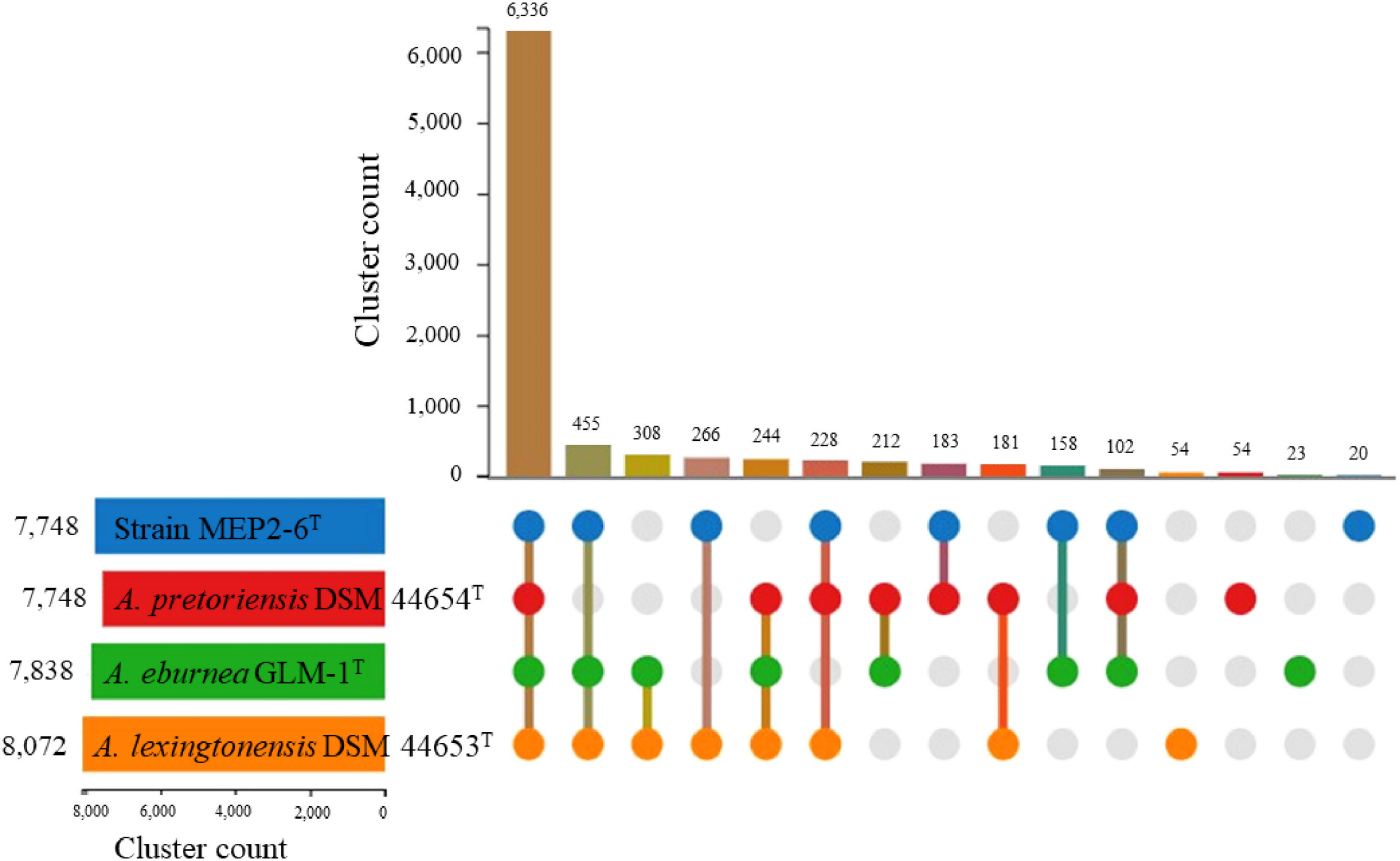
Proteome comparison of strain MEP2-6^T^ and closely related type strains of the genus *Amycolatopsis* based on OrthoVenn3. An UpSet plot showed unique and shared orthologous clusters among species. The left horizontal bar chart depicts the number of orthologous clusters per species, whereas the right vertical bar chart illustrates the number of orthologous clusters shared among the species.

To characterize core and strain*-*specific genes, orthologous groups were determined using the translated proteomes of strain MEP2-6^T^ compared to those of the three closest *Amycolatopsis* species: *A. lexingtonensis* DSM 44653^T^, *A. eburnea* GLM-1^T^, and *A. pretoriensis* DSM 44654^T^ (**Figure 5**). In total, 8,824 orthologous clusters and 39,478 proteins were identified. The core genome shared by the four strains was depicted by 6,336 orthologous clusters. In a pairwise comparison, the largest number of orthologous clusters was found for *A. eburnea* GLM-1^T^/*A. lexingtonensis* DSM 44653^T^ (308), followed by strain MEP2-6^T^/*A. lexingtonensis* DSM 44653^T^ (266), *A. eburnea* GLM-1^T^/*A. pretoriensis* DSM 44654^T^ (212), MEP2-6^T^/*A. pretoriensis* DSM 44654^T^ (183), and strain MEP2-6^T^/*A. eburnea* GLM-1^T^ (158). These findings agreed well with the taxonomic position of strain MEP2-6^T^ in the core phylogenomic tree (**Figure 2**).

**Figure 6:**
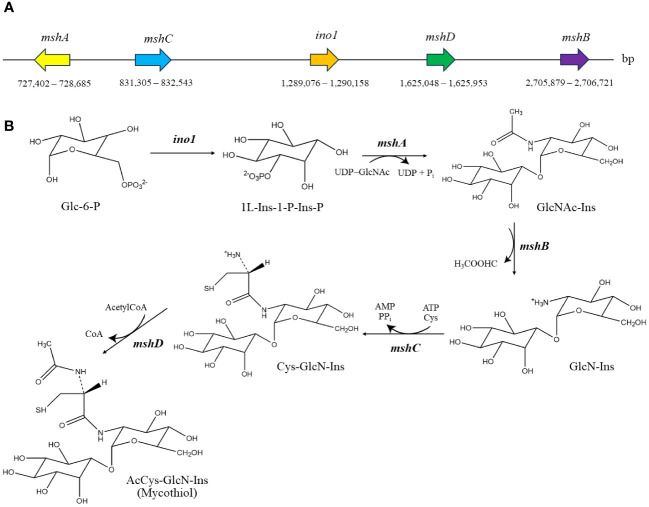
Genomic organization of mycothiol biosynthetic gene cluster **(A)** and biosynthetic pathway of mycothiol **(B)** in strain MEP2-6^T^. The structures were drawn using the ChemDraw program. Glc-6-P, glucose-6-phosphate; 1L-Ins-1-P-Ins-P, 1L-*myo*-inositol-1-phosphate; GlcNAc-Ins, 1-*O*-(2-acetamido-2-deoxy-α-D-glucopyranosyl)-D-*myo*-inositol; GlcN-Ins, 1-*O*-(2-amino-2-deoxy-α-D-glucopyranosyl)-D-*myo*-inositol; Cys-GlcN-Ins, 1-*O*-[2-[[(2*R*)-2-amino-3-mercapto-1-oxopropyl]amino]-2-deoxy-α-D-glucopyranosyl]-D-*myo*-inositol.

As shown in **Figure 5**, the number of strain-specific clusters for each strain was 20 for MEP2-6^T^, 23 for *A. eburnea* GLM-1^T^, 54 for *A. lexingtonensis* DSM 44653^T^, and 54 for *A. pretoriensis* DSM 44654^T^. Strain MEP2-6^T^ uniquely contained an orthologous cluster of the mycothiol biosynthesis process that was not found in the closest relatives. Functional annotation based on the eggNOG and KEGG databases revealed that the strain completely contained all genes encoded for the key enzymes in the biosynthetic pathway of mycothiol: *ino1* (myo-inositol-1-phosphate synthase, EC 5.5.1.4), *mshA* (D-inositol-3-phosphate glycosyltransferase, EC 2.4.1.250), *mshB* (*N*-acetyl-1-D-myo-inositol-2-amino-2-deoxy-alpha-D-glucopyranoside deacetylase, EC 3.5.1.103), *mshC* (L-cysteine:1D-*myo*-inositol-2-amino-2-deoxy-alpha-D-glucopyranoside ligase, EC 6.3.1.13), and *mshD* (mycothiol synthase, EC 2.3.1.189). The protein sequences of the genes encoding mycothiol biosynthetic enzymes showed sequence identities ranging from 94.0% to 98.9% to the reference protein based on UniProt BLAST (Coudert et al., 2023). Proteins with over 90% sequence identity typically share the same biological processes (Joshi and Xu, 2007). The organization of the gene cluster and biosynthetic pathways is illustrated in **Figure 6**.

### Predictive functional signatures to live inside plant

Based on RAST annotation and enzyme prediction by KEGG, a plant-derived strain MEP2-6^T^ illustrated an important enrichment of genes related to encoding enzymes for plant-synthesized sugar interconversions, which were absent in *A. eburnea* GLM-1^T^ isolated from arbuscular mycorrhizal fungi and *A. lexingtonensis* DSM 44653^T^ and *A. pretoriensis* DSM 44654^T^ isolated from equine placentas. These genes included *galA*, *xynD*, *cel74a*, *cbhA*, and *bglB*-*bglX*, which encode α-galactosidase (EC 3.2.1.22), arabinoxylan arabinofuranohydrolase (EC 3.2.1.55), xyloglucan-specific exo-β-1,4-glucanase (EC 3.2.1.155), cellulose-1,4-beta-cellobiosidase (exoglucanases, EC 3.2.1.91), and β-D-glucosidases (EC 3.2.1.21), respectively. The genes *malZ*, *chiA*, and one encoded maltodextrin glucosidase (EC 3.2.1.20), chitinase (E 3.2.1.14), and endoglucanase (EC 3.2.1.4), respectively, were found in all strains. Nonetheless, the gene coding for endoglucanase was over-represented in strain MEP2-6^T^ with seven copies.

Several transporters of oligo- and monosaccharides were overexpressed in strain MEP2-6^T^. ABC transporter genes for various sugars, such as chitobiose (*dasA*, *dasB*, and *dasC*), raffinose/stachyose/melibiose (*msmE*, *msmF*, *msmG*, *msmX*, and *msmK*), fructose (*frcC*, *frcB*, and *frcA*), trehalose/maltose (*thuE*, *thuF*, and *thuG*), and multiple sugars (e.g., *malK*, *sugC*, and *msiK*), were found to be more than or equal to a two-fold copy among the closest non-plant-associated *Amycolatopsis* species. The ABC transporter genes *cebE*, *cebF*, and *cebG*, which encode the cellobiose transport system, were only present in strain MEP2-6^T^. Interestingly, strain MEP2-6^T^ takes up fructose into the cell via the fructose ABC transport system, but the three closest species, *A. lexingtonensis* DSM 44653^T^, *A. eburnea* GLM-1^T^, and *A. pretoriensis* DSM 44654^T^, ingest fructose into the cells through the phosphotransferase system (PTS) encoded by four genes: *fruA*, *fruB*, *fruK*, and *ptsI*. These genes located in the genome of strain MEP2-6^T^ prove that the strain can import and utilize various sugars as sole carbon sources.

The amino acids excreted by the host plant can serve as nitrogen sources for plant-derived actinobacteria. Genes associated with isoleucine, leucine, and valine were distributed in both plant-associated and non-plant-associated actinobacteria. However, it is fascinating that genes encoding branched-chain amino acid transporters (*livF*, *livG*, *livH*, and *livM*) were found to be overexpressed in strain MEP2-6^T^, with over two copies per genome. As an insight into the stress response, all strains employed a similar system, for instance, glutathione peroxidase (*gpx*, EC 1.11.1.9) and superoxide dismutase Fe–Mn family (*sod2*, EC 1.15.1.1). Interestingly, only strain MEP2-6^T^ (plant-associated) contained the superoxide dismutase Cu–Zn family (*sod1*, EC:1.15.1.1) and rubredoxin, which are utilized for its ability to respond to oxidative stress.

Protein secretion plays a crucial role in modulating bacteria–niche interactions, particularly in the symbiotic (parasitic, mutualistic, or commensal) colonization of bacteria. In Gram-positive bacteria, secretion proteins are exported out of the cytoplasm by the conserved Sec translocase system, twin-arginine translocation (TAT) system, or, alternately, by the type VII system (Tseng et al., 2009). The genome of strain MEP2-6^T^ encodes 1,050 (11.3%) secreted proteins involved in several protein secretion systems (**Table 5**). The strain comprises all genes responsible for coding the protein-conducting channel SecYEG, the ATP-dependent motor protein SecA, and the ancillary membrane protein complex SecDF, which delivers secretory proteins across the plasma membrane through the translocase (Lycklama et al., 2012). In addition, strain MEP2-6^T^ contained all genes, *tatA*, *tatB*, and *tatC*, which encode twin-arginine translocation proteins that transport folded proteins across the plasma membrane (Palmer and Berks, 2012) and included four genes as part of the type VII secretion system: *eccB*, *eccC*, *eccD*, and *mycP* to promote host colonization.

**Table 5:** Genes responsible for encoding protein secretion systems present in the genome of strain MEP2-6^T^.

Secretion system	Gene	Product
**Sec translocase**	*secA*	Preprotein translocase subunit SecA
	*secD*	Preprotein translocase subunit SecD
	*secE*	Preprotein translocase subunit SecE
	*secF*	Preprotein translocase subunit SecF
	*secY*	Preprotein translocase subunit SecY
	*secG*	Preprotein translocase subunit SecG
	*yidC*	YidC/Oxa1 family membrane protein insertase
	*yajC*	Preprotein translocase subunit YajC
	*ffh*	Signal recognition particle subunit SRP54
	*ftsY*	Fused signal recognition particle receptor
	*lspA*	Signal peptidase II
**Twin-arginine translocation**	*tatA*	Sec-independent protein translocase protein TatA
	*tatB*	Sec-independent protein translocase protein TatB
	*tatC*	Sec-independent protein translocase protein TatC
	*tatD*	TatD DNase family protein
**Type II (T2SS)**	*tadA*	TadA family conjugal transfer-associated ATPase
	*tadB*	Tight adherence protein B
**Type VII (T7SS)**	*eccB*	Membrane protein EccB
	*eccC*	FtsK/SpoIIIE family protein
	*eccD*	Integral membrane protein EccD
	*mycP*	S8 family serine peptidase (mycosin-1)

### Diversity of secondary metabolite biosynthetic gene clusters

The genomes of the strain MEP2-6^T^ and its closest *Amycolatopsis* species were evaluated for candidate secondary metabolite biosynthetic gene clusters (BGCs) using antiSMASH 7.0, a pipeline for secondary metabolite identification. The genome of *S. scabiei* 87.22^T^ (accession no. FN554889) was also compared to prove that strain MEP2-6^T^ had no BGCs association in causing scab disease in potatoes. The number of identified BGCs per species, based on antiSMASH, ranged from 27 to 33. Strain MEP2-6^T^ comprised 33 BGCs exhibiting different similarities to gene clusters, with known functions ranging from 4% to 100%. The BGCs that exhibited ≥50% homology to known functional gene clusters are shown in **Table 6**. Based on the antiSMASH version 7.0 annotation, *S. scabiei* 87.22^T^, which is a well-known causative agent of potato scab disease, contained BGCs encoded for the phytotoxins associated with the occurrence of scab lesions on potato tubers, including thaxtomin, bottromycin, and concanamycin A (**Supplementary Table 2**) (Li et al., 2019a). To prove that strain MEP2-6^T^ is not a potato scab-causing pathogen, the protein sequences of the gene clusters responsible for synthesizing thaxtomin, bottromycin, and concanamycin A from *S. scabiei* 87.22^T^ were subjected to a BLASTP search against all proteins of strain MEP2-6^T^. S. *scabiei* 87.22^T^ comprised 52, 17, and 54 protein-coding genes in the BGCs of thaxtomin, bottromycin, and concanamycin A, respectively. Strain MEP2-6^T^ contained 7, 2, and 25 protein-coding genes for the thaxtomins, bottromycin, and concanamycin A BGCs, respectively. Although protein-coding genes related to the BGCs of thaxtomin, bottromycin, and concanamycin A were present, the strain could not produce the compounds because those of the protein-coding genes were not the core biosynthetic gene clusters. For instance, two genes clustered in bottromycin BGC and found in strain MEP2-6^T^ were identified as *btmA*. This gene encodes the phosphotransferase system (PTS) transporter subunits EIIC and IIE (Franz et al., 2021), which are responsible for selecting and transporting sugar molecules across the bacterial cytoplasmic membrane (McCoy et al., 2015). According to the seven protein-coding genes of strain MEP2-6^T^ that are associated with thaxtomin BGC, two significant genes were identified as *txtD* and *txtH*, which encode nitric oxide synthase and MbtH family non-ribosomal peptide synthase (NRPS) accessory proteins, respectively. The *txtH* gene functions as a chaperone by promoting proper folding and stimulation of the two crucial NRPS enzymes encoded by *txtA* and *txtB* (Li et al., 2019b). However, strain MEP2-6^T^ was unable to produce thaxtomin because it had no *txtA*, *txtB*, or *txtC*, which are the core biosynthetic genes responsible for catalyzing the conversion of L-tryptophan to thaxtomin (Jiang et al., 2018). Based on the 25 protein-coding genes of strain MEP2-6^T^ related to the BGC of concanamycin A, four significantly encoded type I polyketide synthases and two encoded acyl carrier proteins (ACP). Nonetheless, it could not produce concanamycin A because it lacks the core synthesis domains of ketosynthase (KS) and acyltransferase (AT) (Haydock et al., 2005). Consequently, based on the analysis of the BGCs, it can be concluded that strain MEP2-6^T^ is not a phytopathogen, even though it inhabits the scab lesions of potato.

**Table 6:** Distribution of identified biosynthetic gene clusters (≥50% homology with known BGCs) encoding for secondary metabolites in strain MEP2-6^T^.

Region	BGC Type	Position (bp)		Most Similar Known Cluster	Similarity^a^	Chemical Class
		From	To			
**1**	Lanthipeptide-class-iii	816,336	838,879	Ery-9/Ery-6/Ery-8/Ery-7/Ery-5/Ery-4/Ery-3 (Erythreapeptin)	100	RiPP: Lanthipeptide
**2**	Ectoine	2,040,835	2,051,227	Ectoine	100	Other
**6**	T1PKS	3,332,994	3,459,247	Tetrafibricin	100	Polyketide + Other
**7**	Thiopeptide, T1PKS, oligosaccharide	3,755,483	3,879,105	Amycolamycin A/Amycolamycin B	51	Polyketide
**11**	Terpene	4,502,482	4,522,010	Isorenieratene	71	Terpene
**17**	NRP-metallophore, NRPS	5,148,968	5,212,319	Scabichelin	80	NRP
**23**	Betalactone, terpene	6,665,558	6,707,896	2-methylisoborneol	100	Terpene
**26**	T1PKS	7,394,059	7,582,465	Candicidin	52	NRP + Polyketide
**30**	NRPS, nucleoside	9,087,404	9,139,570	Detoxin P1/Detoxin P2/Detoxin P3	100	NRPS + Polyketide
**31**	Terpene	9,554,549	9,573,112	Geosmin	100	Terpene
**33**	NAPPA	10,223,959	10,257,383	ϵ-Poly-L-lysine	100	NRP

a Similarity is the fraction of homologous genes in the query and the hit clusters. NAPAA, non-alpha poly-amino acids like ϵ-poly-lysine; Nl-siderophore, NRPS-independent, IucA/IucC-like siderophores; NRPS, non-ribosomal peptide synthetase; NRPS-like, NRPS-like fragment; PKS-like, other types of polyketide synthase; T1PKS, type I polyketide synthase; T2PKS, type II polyketide synthase; T3PKS, type III polyketide synthase; hglE-KS, heterocyst glycolipid synthase-like PKS; Other, cluster containing a secondary metabolite-related protein that does not fit into any other category.

**Figure 7:**
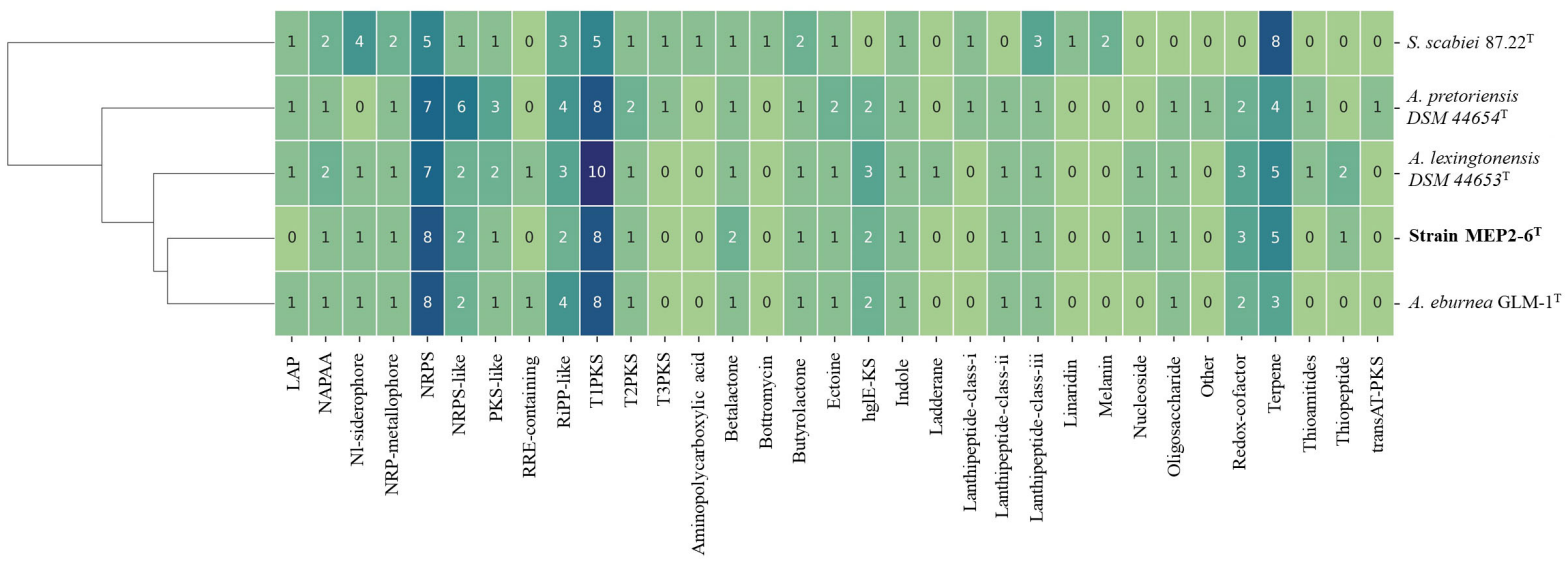
Distribution of BGCs across the genome of strain MEP2-6^T^ and its closest relatives. The hierarchical heatmap depicts the number of genes assigned to the individual smBGCs. Rows were clustered using Euclidean distances. LAP, linear azol(in)e-containing peptides; NAPAA, non-alpha poly-amino acids such as ϵ-poly-lysine; Nl-siderophore, NRPS-independent, IucA/IucC-like siderophores; NRPS, non-ribosomal peptide synthetase; NRPS-like, NRPS-like fragment; PKS-like, other types of polyketide synthase; T1PKS, type I polyketide synthase; T2PKS, type II polyketide synthase; T3PKS, type III polyketide synthase; hglE-KS, heterocyst glycolipid synthase-like PKS; Other, cluster containing a secondary metabolite-related protein that does not fit into any other category.

The distribution of secondary metabolite biosynthetic gene clusters (BGCs) among the strains in this study is presented in **Figure 7** as hierarchical clusters. Among the four *Amycolatopsis* species, the three most frequently presented classes of BGCs encode genes for the production of type I polyketide synthases (T1PKS), non-ribosomal peptide synthases (NRPS), and terpenes. It can be determined that the pattern of BGCs is correlated with species phylogeny. Strain MEP2-6^T^, isolated from scab lesions on potato tubers, shared a monophyletic clade with *A. eburnea* GLM-1^T^, isolated from spores of *Funneliformis mosseae* RYA08, an arbuscular mycorrhizal fungus that inhabits *Aquilaria crassna* Pierre ex Lec (Chaiya et al., 2019) and a polyphyletic clade with *A. lexingtonensis* DSM 44653^T^ isolated from lesions on horse placentas (Labeda et al., 2003). This phylogenetic cluster was consistent with the genomic similarity and the core phylogenomic tree. Although the BGCs seemed to be correlated based on species phylogeny, some BGCs and their products were different among the four *Amycolatopsis* strains; for example, ladderane and thioamitide BGCs were not found in strain MEP2-6^T^ and *A. eburnea* GLM-1^T^ but were present in *A. lexingtonensis* DSM 44653^T^ and *A. pretoriensis* DSM 44654^T^. Compounds encoded by BGCs (≥50% homology with known functions) were found in all and/or were unique in the four *Amycolatopsis* strains, as shown in **Table 7**.

**Table 7:** Predicted compounds encoded by biosynthetic gene cluster families (GCFs) identified in the genomes of strain MEP2-6^T^ and its closest *Amycolatopsis* species with ≥50% homology with known functions.

Strain	Gene Cluster Families															
	Ectoine	PKS					NRPS		Hybrid PKS-NRPS			Terpene			RiPP	NAPAA
	Ectoine	Tetrafibricin	Amycolamycins	Nystatin	Macrotermycin	Xantholipin	Scabichelin	Limazepines	Candicidin	Detoxins	Arixanthomycins	Geosmin	Isorenieratene	2-Methylisoborneol	Erythreapeptin	ϵ-Poly-L-lysine
MEP2-6^T^																
*A. lexingtonensis* DSM 44653^T^																
*A. eburnea* GLM-1^T^																
*A. pretoriensis* DSM 44654^T^																

Yellow, presence; Light grey, absence.

### Pathogenicity on plant

A tomato seedling test was conducted to confirm the pathogenicity of strain MEP2-6^T^, and the findings are shown in **Supplementary Figure 6**. Tomato seeds cultured with strain MEP2-6^T^ germinated as expected, whereas those cultured with *S. scabiei* WSLK1-9 did not germinate. Moreover, the potato tuber slice test revealed that strain MEP2-6^T^ could not necrotize tissues on potato tuber slices compared to the control *S. scabiei* WSLK1-9. Thus, it can be concluded that strain MEP2-6^T^ is non-phytopathogenic and unrelated to the cause of potato scab disease.

### Endophytic colonization of *Solanum tuberosum* L. cv. Spunta by strain MEP2-6^T^

**Figure 8:**
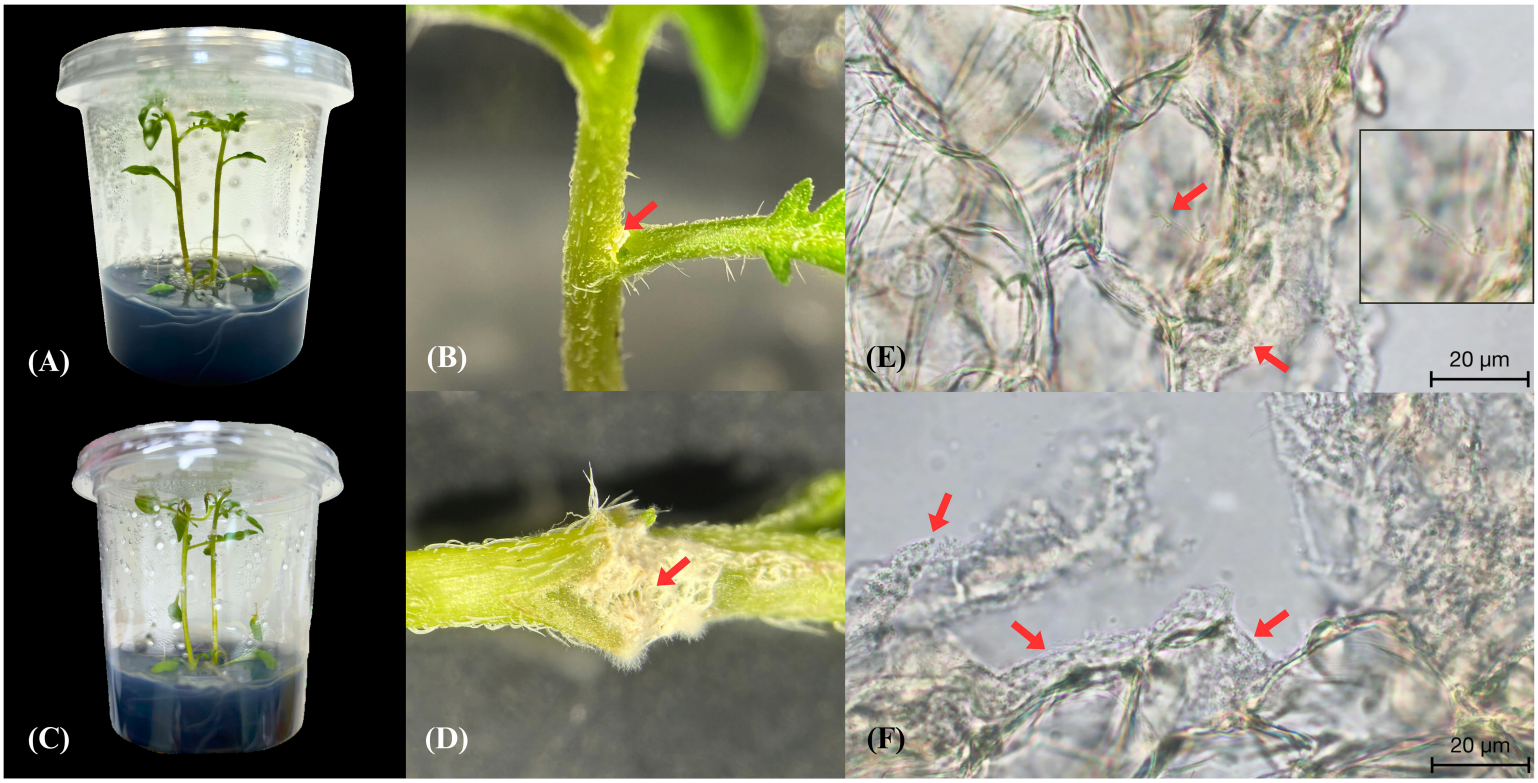
Endophytic colonization of *Solanum tuberosum* L. cv. Spunta by the *Amycolatopsis* strain MEP2-6^T^. The potato culture before inoculation of strain MEP2-6^T^ **(A)**, hyphae with spore masses of strain MEP2-6^T^ inoculated into the stem node wounding site **(B)**, the normal potato culture after inoculation of strain MEP2-6^T^ for five days **(C)**, attachment of white mycelia of strain MEP2-6^T^ on the stem node **(D)**, sections of *S. tuberosum* cv. Spunta stem epidermal cells invaded by *Amycolatopsis* MEP2-6^T^ **(E, F)**. Scale bar 20 μm. The boxed part of the image is shown as a magnification on the right side of **(E)**. Red arrows indicate white mycelia in **(B, D)** and indicate vegetative cells in **(E, F)**.

As strain MEP2-6^T^ encompassed genes related to niche colonization in its genome, we examined where it enters the endosphere of *S. tuberosum* L. cv. Spunta to gain insights into the endophytic biology of the genus *Amycolatopsis*. Consequently, we inoculated mycelia with spore masses of *Amycolatopsis* strain MEP2-6^T^ into the wounding stem node site of 14-day-old *S. tuberosum* L. cv. Spunta cultures (**Figures 8A, B**). At 5 days post-inoculation, the potato culture grew regularly (**Figure 8C**), and strain MEP2-6^T^ attached to the stem node in dense white mycelia (**Figure 8D**) without damage. Colonized *S. tuberosum* L. cv. Spunta stems were then excised using a free-hand-cut technique with a fine razor blade for sectioning and visualization with high-resolution bright-field microscopy. The findings showed that strain MEP2-6^T^ colonized not only the stem surface but also the internal stem tissue and, notably, the intracellular space (**Figures 8E, F**). Incredibly, no plant cellular membrane separates *Amycolatopsis* from its intracellular space.

### Production of extracellular carbohydrate-degrading enzymes

**Figure 9:**
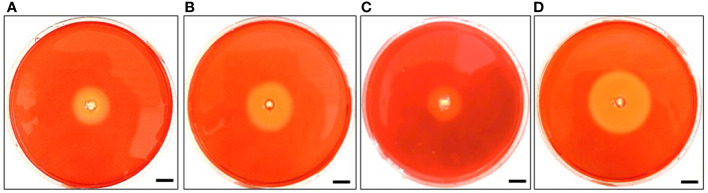
Diameter of the clear transparent zone illustrating endoglucanase activity of strain MEP2-6^T^ and its closest type strains. Strain MEP2-6^T^ **(A)**, *A*. *pretoriensis* JCM 12673^T^ **(B)**, *A*. *lexingtonensis* JCM 12672^T^ **(C)**, and *A*. *eburnea* TBRC 9315^T^ **(D)**. Scale bar 10 mm.

The genomes of strain MEP2-6^T^ and its closest type strains exhibit many genes encoding enzymes potentially involved in carbohydrate-degrading enzymes, particularly cellulose-binding related genes. Genes encoding endoglucanases and chitinases were observed in strain MEP2-6^T^ and its closest relatives, whereas *cbhA* and bglB-*bgl*X encoded exoglucanases and *β*-D-glucosidases, respectively, were uniquely detected in strain MEP2-6^T^. These results clearly indicate that all strains could hydrolyze cellulose. Nevertheless, the symbiotic actinobacterium *Frankia* sp. has reduced a set of carbohydrate-degrading enzyme genes in its genome, especially pectinase (Pujic et al., 2012). Strain MEP2-6^T^ and its closest neighbors were examined for extracellular production of endoglucanases, pectinases, and chitinases. All strains produced endoglucanases by observing the clear transparent zone on carboxymethyl cellulose (CMC) agar (**Figure 9**) but did not produce pectinase and chitinase (**Supplementary Table 3**).

## Discussion

Recently, the integration of polyphasic taxonomy and genome sequence-based taxonomy has provided precision, reliability, and reproducibility for bacterial classification (Nouioui et al., 2018). In the present study, we unambiguously identified a novel endophytic actinomycete species, strain MEP2-6^T^, isolated from scab lesions on potato tubers. Sequence analysis of the 16S rRNA gene revealed that strain MEP2-6^T^ belonged to the family *Pseudonocardiaceae*, order *Pseudonocardiales*, class *Actinomycetia*, and phylum *Actinomycetota.* Results from chemotaxonomic characteristics, including a type IV cell wall, a type A whole-cell sugar pattern (Lechevalier et al., 1971), and a type PII phospholipid type II (Lechevalier et al., 1977), also indicated that the strain was a member of the genus *Amycolatopsis*. Based on a combination of 16S rRNA gene phylogenetic and core phylogenomic analyses (**Figures 1**, **2**), strain MEP2-6^T^ distinctly shared the closest relationship with *A. lexingtonensis* NRRL B-24131^T^ (=JCM 12672^T^ =DSM 44653^T^), *A. pretoriensis* DSM 44654^T^ (=JCM 12673^T^), and *A. eburnea* GLM-1^T^ (=TBRC 9315^T^). ANIb, ANIm, and dDDH values were used to confirm the novelty of strain MEP2-6^T^. The values of the two types of ANI and dDDH between the strain and its closest strains (**Table 4**) were significantly below the cut-off values recommended for species delineation: <95% for ANIb, <96% for ANIm (Richter and Rosselló-Móra, 2009), and <70% for dDDH (Wayne et al., 1987; Goris et al., 2007). Thus, it should be noted that strain MEP2-6^T^ represents a new species within the genus *Amycolatopsis*, for which the name *Amycolatopsis solani* sp. nov. was proposed.

In this study, no significant correlation was observed between genome size and environment. The genome sizes of plant-derived *Amycolatopsis* strain MEP2-6^T^ and its closest *Amycolatopsis* species were very similar (10.2 Mb ± 0.5 Mb). This finding is in contrast to that of *Kitasatospora* sp. SUK42, which occupies the stems of *Antidesma neurocarpum* Miq, adapted to an endophytic lifestyle via genome reduction (Zin et al., 2021). On one hand, the genomic reduction did not occur in all endophytic actinobacterial genera. *Micromonospora* and *Streptomyces* appeared to have developed so as to adapt to multiple ecological niches, which could be altered to larger genomes to shelter different lifestyles (Trujillo et al., 2014; Quach et al., 2022; Zhou et al., 2023).

Identifying orthologous clusters is crucial for comparative genomic studies because it allows comparison of evolutionary relationships between genes across different species (Sun et al., 2023). This study illustrates the novel strain MEP2-6^T^ and its closest *Amycolatopsis* species, which shared 6,336 orthologous clusters associated with biological, molecular, and cellular functions (**Figure 5**). Although most of the functions relied on orthologous clusters that were similar among the strains, the function of the mycothiol biosynthesis process was unique in strain MEP2-6^T^. It effectively comprises all the essential genes in the biosynthetic pathway of mycothiol (**Figure 6**). Living within plants of microbial endophytes often induces stress-responsive genes to generate reactive oxygen species (ROS) scavengers (Bosamic et al., 2020). Mycothiol plays a vital role in the detoxification of alkylating agents, reactive oxygen and nitrogen species, and antibiotics and also acts as a thiol buffer, which is crucial for maintaining a highly reducing environment within the cell (Newton et al., 2008). Consequently, strain MEP2-6^T^ was able to live inside the plant tissues.

Living as a plant endophyte, bacteria must have a genetic system to utilize plant-synthesized carbohydrates as a nutritional source (Fabryová et al., 2018; Jiménez-Gómez et al., 2019). Our study revealed that the plant-associated actinobacteria strain MEP2-6^T^ was rich in genes related to encoding plant-synthesized polysaccharide interconversion enzymes: *galA*, *xynD*, *cel74a*, *cbhA*, and *bglB*-*bglX*, which are depleted in *A. eburnea* GLM-1^T^ residing in arbuscular mycorrhizal fungi, *A. lexingtonensis* DSM 44653^T^, and *A. pretoriensis* DSM 44654^T^, occupying the equine placenta. The *xynD* gene encodes arabinoxylan arabinofuranohydrolase (EC 3.2.1.55), which plays a crucial role in the conversion of arabinoxylan to L-arabinose (Lee et al., 2001). Altogether, *cel74a* plays a vital role in encoding xyloglucan-specific exo-β-1,4-glucanase (EC 3.2.1.155), which is responsible for converting polymeric xyloglucan to heptasaccharides (Wong et al., 2010). The genome of strain MEP2-6^T^ uniquely contained the *cbhA* and *bglB*-*bglX* genes encoding exoglucanases and β-D-glucosidases, respectively. These genes are typically found in endophytic actinomycetes such as *Fodinicola acacia*, *Frankia* sp. (Phạm et al., 2020), and *Streptomyces* sp. (El-Gendy et al., 2022). Moreover, the gene encoding endoglucanase (EC 3.2.1.4), which is responsible for randomly cleaving the cellulose polymer into more petite sugar and oligomeric polysaccharides (Rahman et al., 2018), was found to be higher in copies of the strain MEP2-6^T^ than in the closest non-plant-associated *Amycolatopsis* species. These findings are in accordance with those of previous studies, which indicated that *Micromonospora lupini* Lupac 08, *M. noduli* GUI 43, and *M. saelicesensis* Lupac 09 isolated from the root nodules of Leguminosae plants as endophytic actinobacterial models comprised a significant number of functional genes related to plant polysaccharide-degrading enzymes (Trujillo et al., 2014; Trujillo et al., 2007; Riesco et al., 2022). Similar reports have revealed that although plant-derived and non-plant-derived bacterial genomes differ in the presence and absence of functional genes associated with carbohydrate degradation, they are phylogenetically related (Levy et al., 2018; Riesco et al., 2022).

Membrane transport systems are remarkably related to behavior and are intrinsic for a microbe to survive in a given environment (Ren and Paulsen, 2007). Numerous oligo- and monosaccharide transporter systems, including chitobiose, raffinose/stachyose/melibiose, fructose, trehalose/maltose, and multiple sugar transporters, were found to be overexpressed in plant-derived *Amycolatopsis* MEP2-6^T^. These results correlate well with the carbohydrate metabolism of several endophytic bacteria that requires the introduction of sugars released by plants in the form of root exudates into cells to serve as carbon sources (Badri and Vivanco, 2009). Moreover, a recent study showed that several endophytic and rhizosphere species of the genus *Pseudomonas* responded to root exudates by inducing several transport systems that encode a Major Facilitator Superfamily (MFS) transporter (Mavrodi et al., 2021). In the present study, the cellobiose transporter was individually expressed in the plant-derived *Amycolatopsis* strain MEP2-6^T^. This transporter is typically found in *Sinorhizobium* and *Rhizobium*, root nodule endosymbionts (Iyer et al., 2016). Strain MEP2-6^T^ actively ingested fructose into cells via the fructose ABC transporter, while the three closest species used the phosphotransferase system (PTS). This finding is in good agreement with those of previous studies conducted by Lambert et al. (2001) and Pinedo and Gage (2009), who reported that the root nodule, an endosymbiotic *Sinorhizobium meliloti*, lacked transport-related PTS proteins necessary for sugar transport and fructose uptake by the fructose ABC transporter.

Plant-associated and rhizosphere bacteria typically use amino acids released by plants as carbon and nitrogen sources (Riesco et al., 2022). In our study, *liv* genes, encoded as transporters for branched-chain amino acids were overexpressed in the potato tuber-associated strain MEP2-6^T^. Branched-chain amino acids were identified as key factors in the relationship between bacteria and Leguminosae plants by serving as nitrogen sources for the bacteria. Their transporters are necessary to facilitate their movement across the symbiosomal membrane to make nitrogen available to the bacteria (Prell et al., 2009). The transporters of branched-chain amino acids were also abundant in the bacterial community of root colonizers in maize and sugarcane, indicating that the metabolism and transport of amino acids play a critical role in plant–microbe interactions and are not limited to the symbiosis between *Rhizobium* and legumes (de Souza et al., 2019).

As indicated above, in addition to mycothiol, the plant-associated *Amycolatopsis* strain MEP2-6^T^ possesses two major genes, *sod1* and *sod2*, encoded the superoxide dismutase Cu-Zn family and superoxide dismutase Fe-Mn family, respectively, to protect itself from reactive oxygen species produced by the plant’s immune system. This result is in line with that of a previous study, which revealed that the mutualist endophyte *Paraburkholderia phytofirmans* PsJN triggered a weak and temporal defense reaction with an oxidative eruption, and the bacterium protected itself by producing superoxide dismutase (Brader et al., 2017). Our research also revealed that genes encoding rubredoxin were found only in strain MEP2-6^T^. This compound is a non-heme iron protein found in some actinobacterial species of the genera *Mycobacterium*, *Dietzia*, and *Saccharomonospora*, and it plays a critical role in the reduction of superoxide and in the adaptation of plants to changing environmental conditions (Nie et al., 2014; Sushko et al., 2021).

In Gram-positive bacteria, the cell wall peptidoglycan acts as a surface structure for transporting and assembling secretory proteins that interact with the environment, especially the infected host tissues (Schneewind and Missiakas, 2012). Secretory proteins associated with host colonization are exported from the cytoplasmic membrane and interact with the host at the cell wall via the Sec-dependent pathway, Sec-independent twin-arginine translocation (TAT) system, or type VII secretion system (Sutcliffe, 2011). To the best of our knowledge, this is the first analysis of genes related to host plant colonization of the genus *Amycolatopsis*. The *Amycolatopsis* strain MEP2-6^T^ contains four pathways to colonize plant tissues: Sec-dependent, Sec-independent (TAT), T2SS, and T7SS. All genes responsible for encoding membrane protein channel (*secY*, *secE*, and *secG*), ancillary proteins (*secD*, *yidC*, and *yajC*), and the ATPase (*secA*) in the Sec-dependent pathway were found in the strain MEP2-6^T^ genome but lacks *secB* gene for coding the chaperone that targets proteins to the Sec translocon for passage via the plasma membrane (Scott and Barnett, 2006). Although the *secB* gene was absent in the genome of the strain, the fused signal recognition particle receptor encoded by the *ftsY* gene can guide proteins to the translocon for passage through the cytoplasmic membrane (Crowther et al., 2012). Significant genes related to the Sec-independent TAT pathway, including *tatA*, *tatB*, and *tatC*, were also located in the genome of *Amycolatopsis* strain MEP2-6^T^. Like other actinobacteria (i.e., *Frankia* sp. strain CcI3) and other bacteria (e.g., *Vibrio fischeri*), the *tatABC* operon encodes translocase proteins, which play an essential role in the excretion of fully folded proteins across the cytoplasmic membrane via the transmembrane proton gradient as the main driving force for translocation as well as function for host symbiotic colonization (Normand et al., 2007; Dunn and Stabb, 2008). Four genes, *eccB*, *eccC*, *eccD*, and *mycP*, defined as part of the T7SS, were located in strain MEP2-6^T^ genome (**Table 5**). These are organized in the same cluster and involve the crucial proteins for secreting conserved membrane component proteins EccB, EccC, EccD, and S8 family serine peptidase (MycP), which were similar to the T7SS gene cluster of other Gram-positive bacteria: *Mycobacterium*, *Streptomyces*, *Micromonospora*, *Bifidobacterium*, *Bacillus*, and *Streptococcus* (Fyans et al., 2013; Houben et al., 2014; Trujillo et al., 2014; Rivera-Calzada et al., 2021). This secretion system plays an essential role in promoting the colonization of niches and host–microbe interactions between members in *Actinobacteria* and *Firmicutes* (Abdallah et al., 2007; Liu et al., 2023). Two genes, *tadA* and *tadB*, in *Amycolatopsis* strain MEP2-6^T^ were identified as components of the Type II secretion system (T2SS), which encodes the TadA family conjugal transfer-associated ATPase and tight adherence protein B, respectively. Similar to other members of the phylum *Actinobacteria*, *Mycobacterium smegmatis*, *Streptomyces coelicolor*, *Thermobifida fusca*, and *Bifidobacterium breve*, these genes are essential for successful colonization of various environmental niches (Kachlany et al., 2001; Tomich et al., 2007; O’Connell Motherway et al., 2011).

The genome sequences of actinomycetes have a much higher potential for the production of secondary metabolites (Bentley et al., 2002). Based on our insight into the genome of strain MEP2-6^T^, we found fascinating niches of secondary metabolite BGCs, which had the potential to encode metabolites with five major chemical classes: PKS, NRPS, hybrid PKS-NRPS, terpene, and saccharide (**Table 6**). Moreover, the diversity of compounds encoded by BGCs in each strain was different (**Table 7**). Ectoine and scabichelin were ubiquitously detected in all *Amycolatopsis* strains in this study. They play crucial roles in stress protection and iron acquisition (Jones et al., 2019; Richter et al., 2019). The BGC encoding ϵ-poly-L-lysine (ϵ-PL) was also present in the genomes of all the strains. This compound, a homopoly(amino acid) comprised of 25–35 L-lysine residues with amide linkages formed between the *ϵ*-amino and *α*-carboxy groups, is edible, bacteriostatic and non-toxic to humans and the environment. Consequently, they have been extensively used in the food, feed, and pharmaceutical industries as both food and feed preservatives, dietary agents, and gene/drug/vaccine carriers (Wang et al., 2021). BGCs coding for tetrafibricin, a fibrinogen receptor antagonist (Kamiyama et al., 1993), and candicidin, a compound that has the ability to control cucumber *Rhizoctonia* rot (Yao et al., 2021) and inhibit some species of *Rhizopus*, *Mucor*, *Pythium*, *Phytophthora*, *Penicillium*, and *Candida* (Muller, 1958; Jørgensen et al., 2009), were only found in strain MEP2-6^T^. Notably, strain MEP2-6^T^ is a promising biocontrol agent and candidate with strong potential as a novel antibiotic producer. Further studies on this strain are recommended for high-value drug discovery and development.

Secondary metabolite biosynthesis pathways and their associated gene clusters have been determined based on predictions drawn from bioinformatic algorithms and can thereby guide the discovery of interesting compounds (Medema and Fischbach, 2015). However, little is known about the evolution of BGCs, as they are correlated with a species source or phylogeny (Jensen, 2016). According to our work (**Figure 6**), it can be evidently observed that the *Amycolatopsis* strain MEP2-6^T^ and its closest species exhibited a high similarity in their BGC patterns in the hierarchical cluster. The distribution patterns of BGCs were evolutionarily correlated with the species phylogeny. This result is in line with those of studies conducted by Adamek et al. (2018) and Chase et al. (2023), who reported that the BGCs distribution patterns of bacteria were mainly driven by species phylogeny.

*Amycolatopsis* strain MEP2-6^T^ was isolated from the scab tissues on the surface of potato tubers, potato tuber slices, and tomato seedling tests were used to verify its pathogenicity. The strain did not necrotize potato tissue or inhibit tomato seed germination (**Supplementary Figure 6**). This finding is in accordance with the study conducted by Croce et al. (2021), who reported that non-pathogenic actinomycetes had no ability to induce stunting of plant seedlings. At present, evidence suggests that thaxtomins and other secreted phytotoxins, such as bottromycin and concanamycin A, play an important role in the development or severity of potato scab disease rather than other mechanisms (Li et al., 2019a). The genomes of strains MEP2-6^T^ and *S. scabiei* 87.22^T^ were annotated with the antiSMASH 7.0. Protein-coding sequences of strain MEP2-6^T^ were subjected to a BLASTP search against the protein sequences of thaxtomin, bottromycin, and concanamycin A BGCs of *S. scabiei* 87.22^T^. Strain MEP2-6^T^ comprised several protein-coding genes associated with the BGCs of these compounds, yet they are not the core biosynthetic genes; therefore, it was unable to produce three significant phytotoxins: thaxtomin, bottromycin, and concanamycin A (Covington et al., 2021). Based on a combination of the pathogenicity test on plants and the analysis of phytotoxin BGCs, strain MEP2-6^T^ can be regarded as a non-phytopathogenic actinomycete.

Previous studies have revealed colonization of the root surfaces of *Arabidopsis* by actinobacteria (Bulgarelli et al., 2012) and chickpea and sorghum by *Amycolatopsis* strain BCA-696 (Alekhy and Gopalakrishnan, 2016). Moreover, it has been reported that *Streptomyces* strains LUP30 and LUP47B, isolated from lucerne plants, can colonize germinating seeds of wheat (Franco et al., 2017). To the best of our knowledge, the present study is the first to show the presence of a non-streptomycete, *Amycolatopsis*, in *S. tuberosum* L. cv. Spunta stem epidermis cell. Mycelia appeared denser on the plant surface than in the endosphere, which may reflect different physiological characteristics between life outside and inside the plant. *Amycolatopsis* strain MEP2-6^T^ invaded potato stem epidermis cells through minor wounds and lived in vegetative mycelial forms without spore formation. Additionally, potato culture remains asymptomatic. This event agrees with Hallmann et al. (1997) and Rosenblueth and Martínez-Romero (2006), who reported that endophytic bacteria can occupy the plant endosphere during all or part of their life cycle and do not harm the host plant. Endophytic bacteria can be classified as obligate or facultative based on their lifestyle. Obligate endophytes have a complete life cycle in the host plant, and transmission to other plants occurs either vertically or by vectors. In contrast, facultative endophytes have a biphasic life cycle that alternates between plants and soils (Hardoim et al., 2008). Therefore, based on life strategies, *Amycolatopsis* strain MEP2-6^T^ can be assumed to be a facultative endophyte.

Most bacterial endophytes can produce and secrete carbohydrate-degrading enzymes, especially those that are active against cellulose and pectin, to locally disrupt the plant cell wall, facilitate colonization, and spread to other plant parts (Pinski et al., 2019). Based on these results, the strain MEP2-6^T^ and its closest relatives can produce endoglucanase. This enzyme randomly cleaves cellulose polymer into more petite sugars and oligomeric polysaccharides (Menéndez et al., 2015; Rahman et al., 2018). However, no pectinolytic activity was observed. Similar to the findings of the genome analysis, the genes encoding endoglucanase enzymes were present in all strains, while genes related to pectinase production were absent. Although endophytic bacteria colonize host plants via wounds and natural openings such as the stomata and lenticels, endoglucanase activity helps them to colonize successfully; for example, the endophytic *Azoarcus* sp. The BH72 mutant, lacking endoglucanase activity, had a decreased capability to colonize rice roots and could not spread to the plant’s aboveground compartments (Reinhold-Hurek et al., 2006). Endoglucanase activity was also found in plant-symbiotic actinomycetes, *Frankia* AcN14a, *Frankia* Ar112.2 (Igual et al., 2001), and other facultative endophytic actinomycetes, *Micromonospora lupini* Lupac 08 (Trujillo et al., 2014), and *Streptomyces endus* OsiSh-2 (Xu et al., 2017). Strain MEP2-6^T^ and its closest neighbors had no chitinase activity, even though their genomes included the chitinase gene. This phenotype was similar to that of *Bacillus licheniformis* N1, DSM13, and ATCC 14580, in which the chitinase gene in their genomes was silent. This event may be caused by an inactive promoter of the chitinase gene in organisms (Lee et al., 2009).

Here, we report that the potato tuber-derived actinomycete strain MEP2-6^T^ is a novel species of the genus *Amycolatopsis*, whose name was proposed to be *Amycolatopsis solani* sp. nov., and the type of strain is MEP2-6^T^. Comparative genomics reliably provides a better understanding of the underlying genetic mechanisms of the adaptation of *Amycolatopsis solani* MEP2-6^T^ to endophytic lifestyles. Comparative smBGCs exhibited a fascinating genetic potential to synthesize different chemical classes of bioactive secondary metabolites and undoubtedly indicated that the distribution patterns of smBGCs are mainly related to species phylogeny. Moreover, strain MEP2-6^T^ can produce endoglucanase, which is an important enzyme in plant-based biofuels and food-feed industries (Behera et al., 2017), and is also a critical enzyme that inhibits *Phytophthora infestans*, a causative agent of potato late blight disease (Zhang et al., 2023). Thus, we suggest that *A. solani* MEP2-6^T^ could be further investigated as a promising candidate for the discovery of novel bioactive compounds, biotechnological applications, and potato probiotics.

### Description of *Amycolatopsis solani* sp. nov.

*Amycolatopsis solani* (so.la′ni. L. gen. n. *solani* of *Solanum*, the generic name of potato).

A gram-positive, aerobic, mesophilic endophytic actinomycete produced septal substrate and aerial mycelia that fragmented into rod-like elements (0.4–0.5 µm × 1.1–1.4 µm in size). Moderate orange-colored substrate mycelia and pale orange yellow aerial mycelia were well-developed on ISP 2 agar medium. Diffusible pigments were not produced in any of the agar media. Good growth on ISP 2, ISP 3, ISP 4, and ISP 7; moderate growth on ISP 5 and ISP 6; poor growth on nutrient agar medium. Growth occurred at 15°C–37°C (optimal at 30° C), pH 5–9 (optimal at 7) and was tolerated up to 4% (*w/v*) NaCl. Amygdalin, L-arabinose, D-fructose, D-galactose, D-glucose, D-melezitose, *myo*-inositol, L-rhamnose, D-sucrose, and D-xylose were used as sole carbon sources. The acids were produced only from D-sucrose. Coagulation and peptonization of milk, nitrate reduction, and gelatin liquefaction were all positive. Starch hydrolysis and H_2_S production tests were negative. Importantly, α-chymotrypsin, cystine arylamidase, leucine arylamidase, naphthol-AS-BI-phosphohydrolase, and valine arylamidase were positive, whereas acid phosphatase, α-fucosidase, and *N*-acetyl-*β*-glucosaminidase were weakly positive. Tests for alkaline phosphatase, esterase (C4), esterase lipase (C8), α-glucosidase, α-mannosidase, trypsin, α-galactosidase, β-galactosidase, β-glucosidase, β-glucuronidase, and lipase (C14) were negative. Cell wall peptidoglycan is composed of *meso*-diaminopimelic. Whole-cell sugars include arabinose, galactose, glucose, and ribose. The *N*-acyl group of muramic acid in peptidoglycan is an acetyl group. No mycolic acid was detected. The polar lipid profile consists of diphosphatidylglycerol, phosphatidylglycerol, phosphatidylethanolamine, hydroxyphosphatidylethanolamine, an unidentified aminophospholipid, six unidentified phospholipids, an unidentified glycolipid, and five unidentified lipids. MK-9(H_6_) and MK-9(H_4_) are the major and minor menaquinones, respectively. The predominant fatty acids are iso-C_16:0_ and iso-C_15:0._

The type of strain, MEP2-6^T^ (=JCM 36309^T^ =TBRC 17632^T^ =NBRC 116395^T^), was isolated from single lesions at the borders between healthy and scab tissues of surface-sterilized potato tubers collected from Chiang Mai Province, Thailand. The DNA G + C content of the type strain calculated from the genome sequence was 71.7 mol%.

## Data availability statement

The datasets presented in this study can be found in online repositories. The names of the repository/repositories and accession number(s) can be found in the article/**Supplementary Material**.

## Author contributions

TW: Data curation, Formal analysis, Funding acquisition, Investigation, Methodology, Writing – original draft, Writing – review & editing. WM: Investigation, Methodology, Software, Validation, Writing – review & editing. PM: Data curation, Formal analysis, Investigation, Writing – review & editing. NN: Formal analysis, Writing – review & editing. JS: Formal analysis, Writing – review & editing. NC: Formal analysis, Writing – review & editing. SU: Formal analysis, Writing – review & editing. ST: Resources, Software, Validation, Writing – review & editing. NS: Conceptualization, Resources, Supervision, Writing – review & editing. YA: Formal analysis, Writing – review & editing. NK: Conceptualization, Formal analysis, Funding acquisition, Methodology, Project administration, Resources, Software, Supervision, Validation, Writing – original draft, Writing – review & editing.
