# Supplementary material for: Comparative genomics reveals insight into the phylogeny and habitat adaptation of novel Amycolatopsis species, an endophytic actinomycete associated with scab lesions on potato tubers
Source: Front Plant Sci. 2024 Mar 27;15:1346574. doi: 10.3389/fpls.2024.1346574 (PMC11004387; doi:10.3389/fpls.2024.1346574)
Supplement: Supplementary file 1 [file DataSheet_1.pdf]

Comparative Genomics Reveals Insight into the Phylogeny and Habitat Adaptation of *Amycolatopsis solani* sp. nov., an Endophytic Actinomycete Associated with Scab Lesions on Potato Tubers

Thippawan Wannawong[1], Wuttichai Mhuantong[2,3], Pipat Macharoen[1], Nantawan Niemhom[4], Jaruwan Sitdhipol[5], Neungnut Chaiyawan[5], Sarinna Umrung[1], Somboon Tanasupawat[6], Nakarin Suwannarach[7,8]*, Yukihiro Asami[9,10], Nattakorn Kuncharoen[1]*

[1] Department of Plant Pathology, Faculty of Agriculture, Kasetsart University, Bangkok 10900, Thailand
[2] Food Biotechnology Research Team, Functional Ingredients and Food Innovation Research Group, National Center for Genetic Engineering and Biotechnology, National Science and Technology Development Agency, Pathum Thani 12120, Thailand
[3] Enzyme Technology Research Team, Biorefinery and Bioproducts Technology Research Group, National Center for Genetic Engineering and Biotechnology, National Science and Technology Development Agency, Pathum Thani 12120, Thailand
[4] Microbiological and Molecular Biological Laboratory, Scientific Instruments Center, School of Science, King Mongkut's Institute of Technology Ladkrabang, Bangkok 10520, Thailand
[5] Biodiversity Research Centre, Research and Development Group for Bio-Industries, Thailand Institute of Scientific and Technological Research, Pathum Thani 12120 Thailand
[6] Department of Biochemistry and Microbiology, Faculty of Pharmaceutical Sciences, Chulalongkorn University, Bangkok 10300, Thailand
[7] Research Center of Microbial Diversity and Sustainable Utilization, Chiang Mai University, Chiang Mai 50200, Thailand
[8] Department of Biology, Faculty of Science, Chiang Mai University, Chiang Mai 50200, Thailand
[9] Graduate School of Infection Control Sciences, Kitasato University, 5-9-1 Shirokane, Minato-ku, Tokyo 108-8641, Japan
[10] Ōmura Satoshi Memorial Institute, Kitasato University, 5-9-1 Shirokane, Minato-ku, Tokyo 108-8641, Japan

*** Correspondence:** fagrnoku@ku.ac.th (N.K.) Tel.: +668-7361-3499 (N.K.); suwan.462@gmail.com (N.S.) Tel.: +668-6512-7518 (N.S.)

Supplementary Figure 1. A potato scabby tuber.

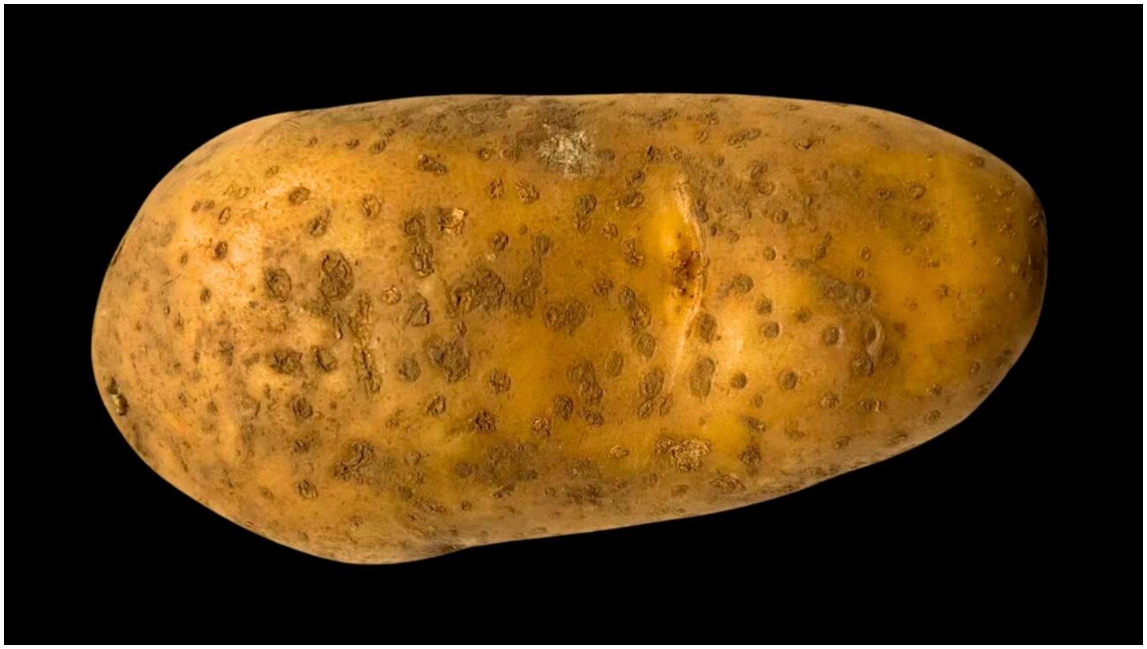

Supplementary Figure 2. Neighbor-joining phylogenetic tree based on the 16S rRNA gene sequences of strain MEP2-6^T and its closely related type strains with validly published names. *Streptomyces scabiei* 87.22^T was used as an outgroup. Bootstrap values ≥ 50% (percentages of 1000 replications) are shown at branch nodes Bar, 0.01 substitutions per nucleotide position.

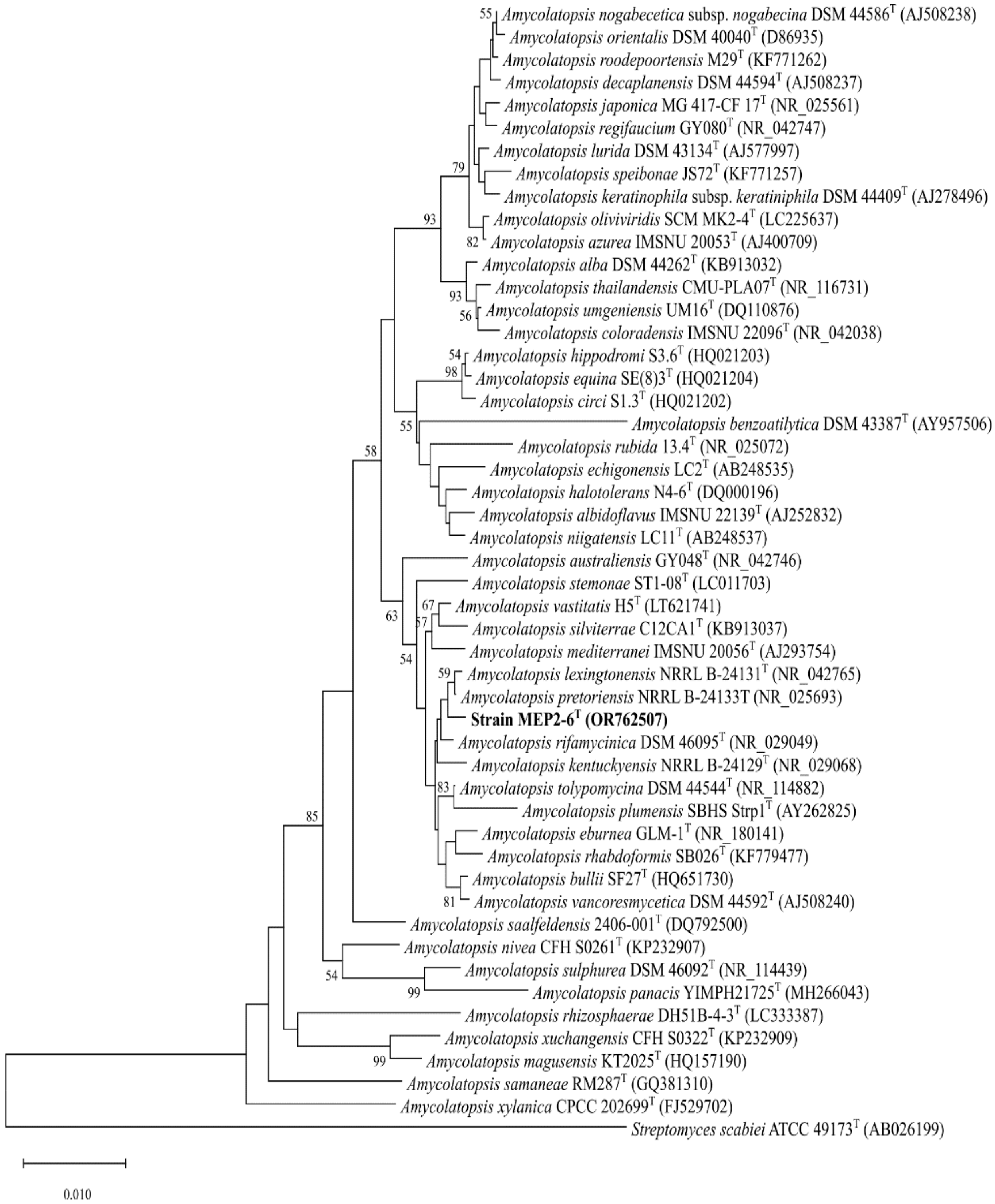

Supplementary Figure 3. Maximum-parsimony phylogenetic tree based on the 16S rRNA gene sequences of strain MEP2-6^T and its closely related type strains with validly published names. *Streptomyces scabiei* 87.22^T was used as an outgroup. The scale bars indicate the phylogenetic distances. Bootstrap values ≥ 50% (percentages of 1000 replications) are shown at branch nodes.

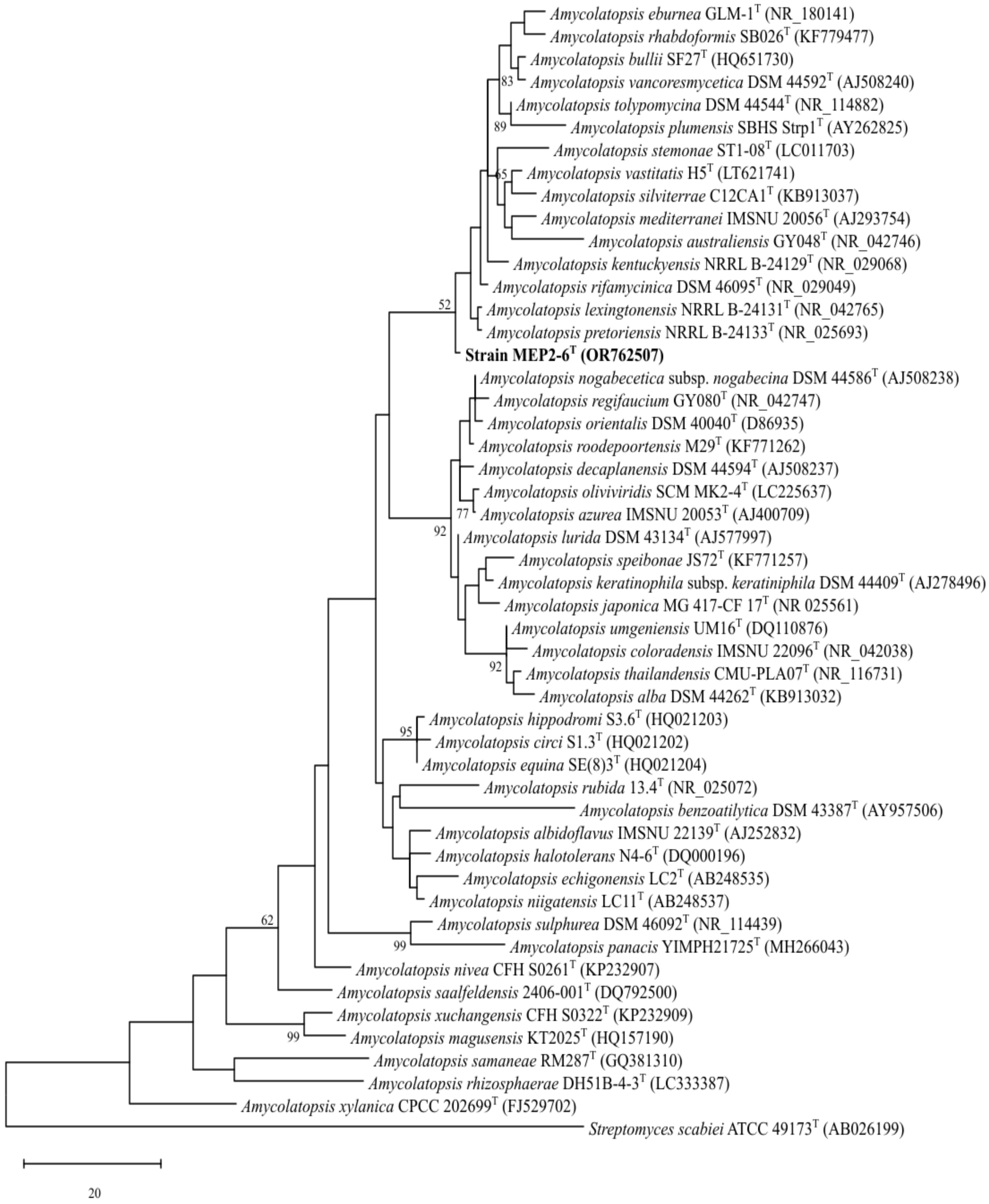

Supplementary Figure 4. Schematic representation of colonial appearances of strain MEP2-6^{T} and its closely related type strains on various agar media. Strain MEP2-6^{T}, *A. lexingtonensis* DSM 44653^{T}, *A. pretoriensis* DSM 44654^{T}, and *A. eburnea* TBRC 9315^{T} on ISP 2 agar, respectively (**1-4**), strain MEP2-6^{T}, *A. lexingtonensis* DSM 44653^{T}, *A. pretoriensis* DSM 44654^{T}, and *A. eburnea* TBRC 9315^{T} on ISP 3 agar, respectively (**5-8**), strain MEP2-6^{T}, *A. lexingtonensis* DSM 44653^{T}, *A. pretoriensis* DSM 44654^{T}, and *A. eburnea* TBRC 9315^{T} on ISP 4 agar, respectively (**9-12**), strain MEP2-6^{T}, *A. lexingtonensis* DSM 44653^{T}, *A. pretoriensis* DSM 44654^{T}, and *A. eburnea* TBRC 9315^{T} on ISP 5 agar, respectively (**1-4**), strain MEP2-6^{T}, *A. lexingtonensis* DSM 44653^{T}, *A. pretoriensis* DSM 44654^{T}, and *A. eburnea* TBRC 9315^{T} on ISP 6 agar, respectively (**13-16**), strain MEP2-6^{T}, *A. lexingtonensis* DSM 44653^{T}, *A. pretoriensis* DSM 44654^{T}, and *A. eburnea* TBRC 9315^{T} on ISP 7 agar, respectively (**17-20**), and strain MEP2-6^{T}, *A. lexingtonensis* DSM 44653^{T}, *A. pretoriensis* DSM 44654^{T}, and *A. eburnea* TBRC 9315^{T} on NA, respectively (**21-24**).

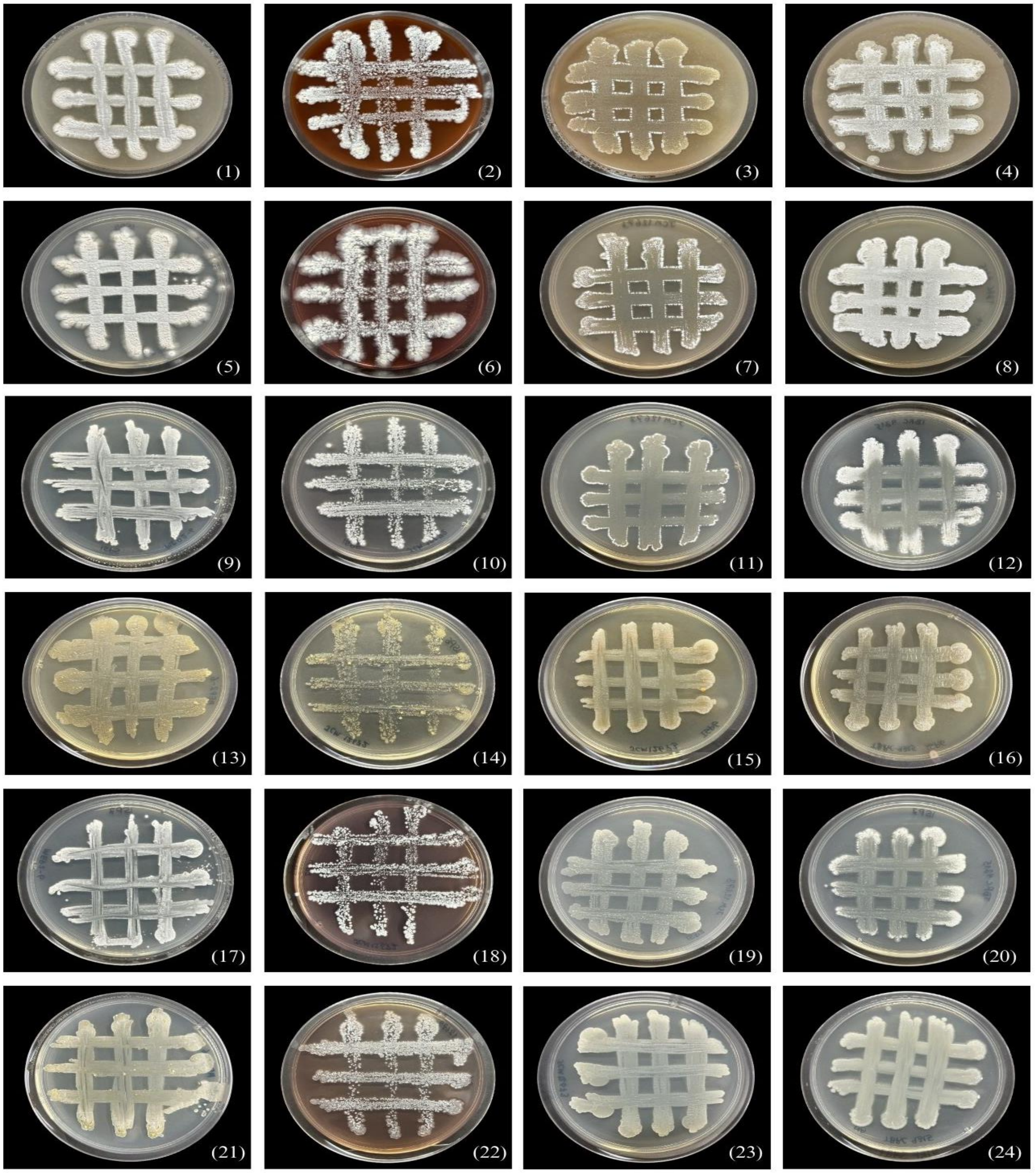

Supplementary Figure 5. Polar lipids profile of strain MEP2-6ᵀ based on a 2-dimensional thin-layer chromatogram detecting with five spraying agents, phosphomolybdic acid, molybdenum blue (Dittmer & Lester reagent), ninhydrin, anisaldehyde, and dragendorff's reagent.
DPG, diphosphatidylglycerol; PG, phosphatidylglycerol; PE, phosphatidylethanolamine; OH-PE, hydroxy-phosphatidylethanolamine; APL, unidentified aminophospholipid; PL1−PL6, unidentified phospholipids; GL, unidentified glycolipid; and L1−L5, unidentified lipids.

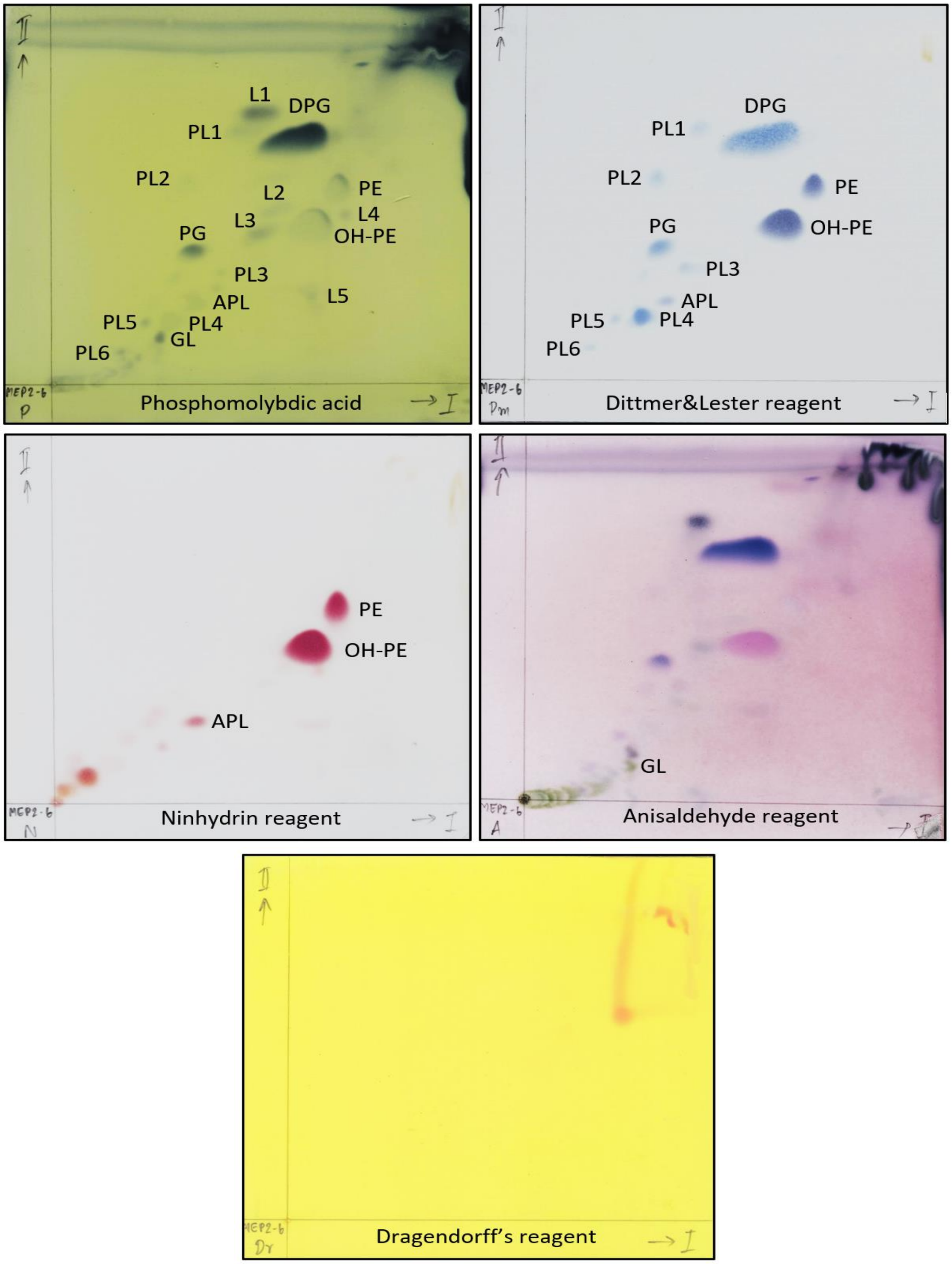

Supplementary Figure 6. Pathogenicity verification of strain MEP2-6^T. Tomato seedling of strain MEP2-6^T (**A**), seedling inoculated with a scab-causing bacteria, *S. scabiei* WSLK1-9 (B), blank control without pathogen of tomato seedling (**C**), potato tuber slice of strain MEP2-6^T (**D**), potato tuber slice with *S. scabiei* WSLK1-9 (**E**), blank control without pathogen of potato tuber slice (**F**).

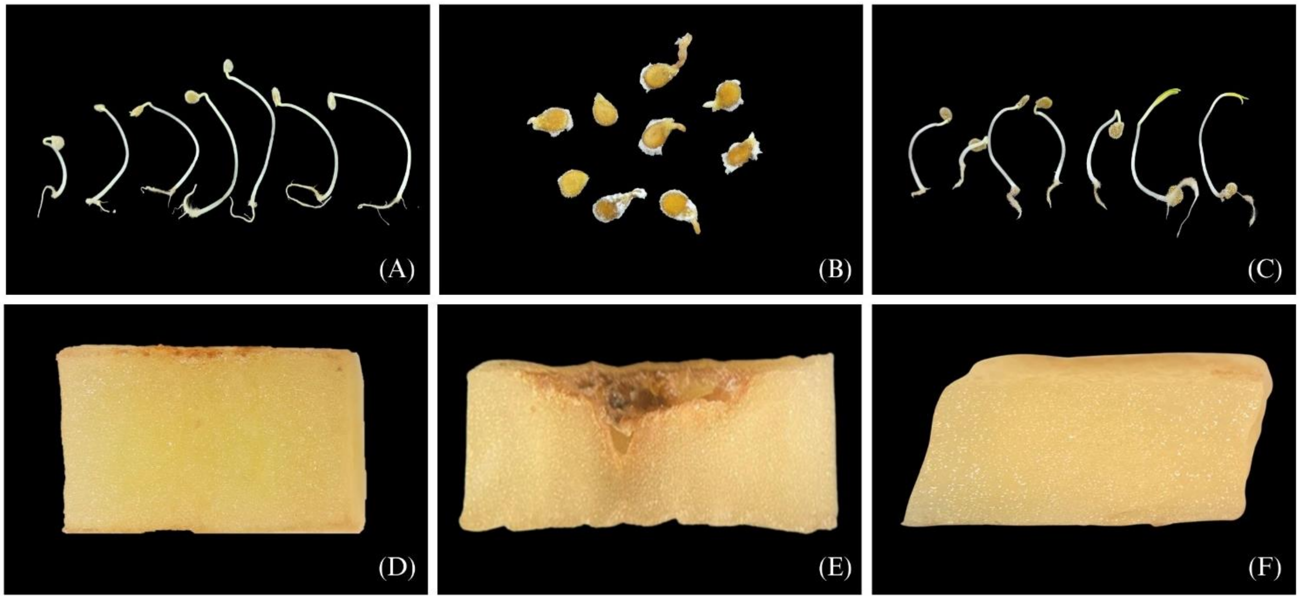

Supplementary Table 1. Cultural characteristics of strain MEP2-6^T and its closely related type strains.

Medium	Strain MEP2-6^T	*A. lexingtonensis* DSM 44653^T	*A. pretoriensis* JCM 12673^T	*A. eburnea* TBRC 9315^T
**ISP medium 2**				
Growth	Good	Good	Good	Good
Spore and aerial mass color	Pale orange yellow	Greenish white	Yellowish white	Yellowish white
Substrate mycelial color	Moderate orange	Dark yellowish brown	Moderate orange yellow	Light yellow
Soluble pigment	None	Very deep red	None	None
**ISP medium 3**				
Growth	Good	Good	Good	Good
Spore and aerial mass color	White	Yellowish white	Yellowish white	White
Substrate mycelial color	Deep orange yellow	Deep yellowish brown	Strong yellow	Pale yellow
Soluble pigment	None	Deep red	None	None
**ISP medium 4**				
Growth	Good	Good	Good	Good
Spore and aerial mass color	White	Greenish white	Yellowish white	White
Substrate mycelial color	Moderate yellow	Dark grayish red	Brilliant yellow	Moderate yellow
Soluble pigment	None	Dark red	None	None
**ISP medium 5**				
Growth	Medium	Good	Good	Medium
Spore and aerial mass color	None	Greenish white	Yellowish white	White
Substrate mycelial color	Moderate yellow	Dark grayish red	Light yellow	Pale greenish yellow
Soluble pigment	None	Very deep red	None	None
**ISP medium 6**				
Growth	Medium	Medium	Medium	Medium
Spore and aerial mass color	Yellowish white	None	None	None
Substrate mycelial color	Moderate orange yellow	Moderate yellow	Moderate yellow	Pale yellow
Soluble pigment	None	None	None	None
**ISP medium 7**				
Growth	Good	Good	Good	Good
Spore and aerial mass color	Yellowish white	Greenish white	White	White
Substrate mycelial color	Brilliant yellow	Dark grayish red	Moderate yellow	Pale yellow
Soluble pigment	None	Very deep red	None	None
**Nutrient agar**				
Growth	Poor	Poor	Poor	Poor
Spore and aerial mass color	None	None	None	White
Substrate mycelial color	Light yellow	Pale yellow	Pale yellow	Yellowish white
Soluble pigment	None	None	None	None

None, no production of mycelial and soluble pigment.

Supplementary Table 2. Distribution of identified biosynthetic gene clusters encoding for secondary metabolites in *Streptomyces scabiei* 87.22^{T}.

Region	BGC Type	Position (bp)		Most Similar Known Cluster	%Similarity[a]	Chemical Class
		From	To			
1	NRP-metallophore, NRPS	124,877	188,045	Thiazostatin/Watasemycin A/Watasemycin B/ 2-Hydroxyphenylthiazoline enantiopyochelin/Isopyochelin	73	NRPS
2	Betalactone	210,499	245,699	Esmeraldin	8	Polyketide + Aminocoumarin
3	NRP	345,318	400,458	Rotihibin A	100	NRPS
4	Lanthipeptide-class-iii	402,211	423,146	Labyrinthopeptin A2/Labyrinthopeptin A1/Labyrinthopeptin A3	60	RiPP: Lanthipeptide
5	Terpene	561,366	581,523	Ebelactone	5	Polyketide
6	Aminopolycarboxylic-acid, terpene	590,921	628,185	EDHA	88	Other
7	Lanthipeptide-class-iii	958,523	980,742	Informatipeptin	100	RiPP: Lanthipeptide
8	Butyrolactone	1,354,553	1,365,035	Lactonamycin	5	Polyketide
9	Terpene	1,445,117	1,469,780	Hopene	92	Terpene
10	Nl-siderophore	2,067,195	2,079,101	Grincamycin	8	Polyketide: Type II polyketide + Saccharide: Hybrid/tailoring saccharide
11	NAPAA	2,216,389	2,250,891	Stenothricin	13	NRP: Cyclic depsipeptide
12	terpene	2,273,305	2,294,341	geosmin	100	Terpene
13	terpene	2,615,998	2,636,835	Leucomycin	13	Polyketide
14	NI-siderophore	2,789,747	2,799,722			
15	NRPS, lanthipeptide-class-i	3,581,513	3,646,644	Thaxtomin D/Thaxtomin A/Thaxtomin C/Thaxtomin B	58	NRP
16	T2PKS	4,831,093	4,903,608	Spore pigment	83	Polyketide
17	T1PKS, NRPS	4,907,595	4,958,875	Niphimycins C-E	6	Polyketide
18	Lanthipeptide-class-iii	5,327,434	5,350,019	TVA-YJ-2	7	RiPP
19	Bottromycin	6,301,273	6,322,136	Bottromycin A2	51	RiPP: Bottromycin
20	Nl-siderophore	6,440,983	6,452,205	Desferrioxamin B/Desferrioxamine E	66	Other
21	Melanin	6,584,191	6,592,550	Istamycin	7	Saccharide
22	T1PKS, butyrolactone, LAP	6,948,648	7,010,078	4-Hexadecanoyl-3-hydroxy-2-(hydroxymethyl)-*2H*-furan-5-one	54	Polyketide

Region	BGC Type	Position (bp)		Most Similar Known Cluster	%Similarity[a]	Chemical Class
		From	To			
23	Ectoine	7,828,037	7,838,435	Ectoine	100	Other
24	NAPAA	8,054,774	8,088,649	ε-Poly-L-lysine	100	NRP
25	Terpene	8,147,349	8,166,320			
26	T1PKS, indole	8,700,909	8,763,617	5-Isoprenylindole-3-carboxylate β-*D*-glycosyl ester	28	Other
27	T1PKS	8,793,628	8,848,502	Lasalocid	9	Polyketide
28	T3PKS	8,870,608	8,911,792	Misaugamycin A/Misaugamycin B	15	NRP
29	Terpene	9,109,516	9,130,190			
30	T1PKS, linaridin	9,288,874	9,413,896	Concanamycin A	89	Polyketide
31	Nl-siderophore	9,430,375	9,443,981	Peucechelin	25	NRP
32	NRP-metallophore, NRPS, melanin	9,526,250	9,591,840	Scabichelin	100	NRP
33	Terpene	9,618,596	9,639,636	Alnumycin A/Alnumycin B/Alnumycin C/Alnumycin P/Prealnumycin/Thalnumycin A/Thalnumycin B/K1115A/1,6-Dihydro-8-propylanthraquinone	6	Polyketide: Type II polyketide

[a] %Similarity is the fraction of homologous genes in the query and the hit clusters. NAPAA, non-alpha poly-amino acids like ε-poly-lysine; Nl-siderophore, NRPS-independent, IucA/IucC-like siderophores; NRPS, non-ribosomal peptide synthetase; NRPS-like, NRPS-like fragment; PKS-like, other types of polyketide synthase; T1PKS, type I polyketide synthase; T2PKS, type II polyketide synthase; T3PKS, type III polyketide synthase; hglE-KS, heterocyst glycolipid synthase-like PKS; Other, cluster containing a secondary metabolite-related protein that does not fit into any other category.

Supplementary Table 3. Diameter of clear transparent zone of each carbohydrate-degrading enzyme.

Strain	Diameter of Clear Transparent Zone (mm)		
	**Endoglucanase**	**Pectinase**	**Chitinase**
Amycolatopsis sp. MEP2-6^T	17.33±0.58	0.00	0.00
A. pretoriensis JCM 12673^T	25.50±0.50	0.00	0.00
A. lexingtonensis JCM 12672^T	13.58±0.63	0.00	0.00
A. eburnea TBRC 9315^T	31.83±0.29	0.00	0.00
